# The role of art knowledge training on aesthetic judgements and executive functions

**DOI:** 10.1098/rsos.240175

**Published:** 2025-02-26

**Authors:** Ionela Bara, Emily S. Cross, Richard Ramsey

**Affiliations:** ^1^ Wales Institute for Cognitive Neuroscience, School of Human and Behavioural Sciences, Bangor University, Bangor, UK; ^2^ Social Brain Sciences Lab, Department of Humanities, Social and Political Sciences, ETH Zurich, Zurich, Switzerland; ^3^ School of Psychology and Neuroscience, University of Glasgow, Glasgow, UK; ^4^ Neural Control of Movement Lab, Department of Health Sciences and Technology, ETH Zurich, Zurich, Switzerland

**Keywords:** art knowledge, training, generalization effects, dose–response, aesthetic judgements, executive functions

## Abstract

The study of how we develop art knowledge can provide valuable insights into the underlying cognitive systems that support expertise and knowledge transfer to new contexts. An important and largely unanswered question is whether art knowledge training impacts subsequent judgements of artworks and executive functions. Across three pre-registered experiments (*N* > 630 total), which used a training intervention and Bayesian regression modelling, we explore whether art knowledge training impacts subsequent judgements of artworks and executive functions. Experiments 1 and 2 revealed an effect of art training on aesthetic judgements for trained but not untrained artworks. These training effects were generalized to unseen artworks produced by the same artist (Experiment 1) or another artist with a similar style (Experiment 2), but not to different art styles. Experiment 2 also showed that with larger training ‘doses’ (>16 minutes), the generalization effects are stronger. Experiment 3 showed invariance of the attentional network to art training versus non-art training, suggesting similar sensitivity of executive functions to different types of training. This work shines new light on the cognitive systems that support learning and generalization of learning to new contexts. Likewise, from an applied perspective, it emphasizes that learning and generalization can occur rapidly with a relatively short (approx. 16 minutes) training video.

## 1. General introduction

People typically acquire art knowledge through a variety of practices ranging from visiting art museums to formal art education, as well as leisure time activities, such as drawing and painting. Visiting art museums, in particular, is one of the most convenient ways to facilitate the acquisition of art knowledge through curatorial art-guided tours, which can also enhance the bond of social communities with cultural heritage [1–3]. However, little is known about how acquired art knowledge affects people’s aesthetic judgements or the cognitive mechanisms that support aesthetic judgements. In the current project, we address three main research questions: (i) in what ways and to what extent does art knowledge impact aesthetic judgements of artworks, as well as generalize to unseen art?; (ii) to what extent are training effects on aesthetic judgements dose-dependent?; and (iii) to what extent are the executive functions of attention affected by acquiring art knowledge? By doing so, we provide novel insight into the way expertise development shapes psychological functioning in the context of art appreciation.

Understanding mechanisms that support learning and expertise development has been a central part of psychological research for decades [4–7]. For example, numerous studies have examined the ways in which motor expertise is acquired as well as the cognitive and brain mechanisms supporting skill acquisition [8–14]. In addition, research has demonstrated that knowledge and skills are acquired most effectively in educational settings [15–17], suggesting that schooling is an efficient method for expertise development.

More recently, researchers have become interested in how experience with different forms of art can shape perception and action. This is an important line of research that shines new light on the plasticity of basic systems [10,18–20]. Here, plasticity refers to training-induced neural and behavioural modifications [21–23]. In addition, this area of investigation has important applied consequences, as art might be used as an intervention tool to improve mental and physical well-being [24–27].

To date, different art forms have been studied in relation to expertise development and its associated neural and behavioural changes. Some research has focused on performing or physical art forms, such as dance, to examine expertise and plasticity in the motor domain. For example, the study of action learning and dance has highlighted that learning by doing versus learning by observing can both facilitate learning new movements and rely on a partially shared set of cognitive and neural systems [28–30].

By contrast, recent research has taken a different approach to study expertise by focusing on visual studio artists, such as painters or sculptors [20,31,32], as well as on visual art knowledge acquisition [33,34]. On one hand, there is evidence to show that trained visual artists outperform non-artists on different behavioural tasks involving visuospatial memory and perception [20,31], which may index an artist’s advantage in creative output and cognition. On the other hand, empirical evidence also suggests that art knowledge development mediates aesthetic judgements of artworks. For example, Van Paasschen *et al.* [34] investigated the impact of art knowledge training on judgements of artworks. The results revealed greater ratings of beauty for art experts compared with non-art experts, whereas affective ratings held constant for both art experts and non-experts, suggesting that art knowledge mediates aesthetic preference of artworks. Together, findings from these studies start to coalesce around questions about the plasticity of underlying cognitive and brain systems, as well as the consequences that follow from developing artistic skills and knowledge. However, this research programme is in its infancy and many questions, naturally, remain understudied.

One key question that is central to learning research concerns the extent to which learning generalizes or transfers to new contexts [35,36]. The generalization of knowledge to novel situations is considered the hallmark of human reasoning, oftentimes conceptualized as relational or analogical reasoning [37]. Analogical reasoning involves similarity judgements that require identification and comparison of similar features between a source and target. Ultimately, the target is better understood if it shares similar and familiar features with the source [38]. In the context of art knowledge, however, little is known about the extent to which art knowledge acquired through training can generalize to new contexts. To date, research work by Boddez *et al*. [39] has revealed that adding positive and negative contextual information to artworks affects the evaluations of not only the featured artworks but also extends to similar artworks, suggesting that at least some levels of generalization can occur to previously unseen but similar artworks.

A second key question concerns the optimal dose for learning and transfer of knowledge to different contexts. Intervention-type research has established the main dose-related characteristics, such as the duration, frequency and amount that collectively determine intervention dose–response [40,41]. However, tailoring an optimal dose for an expected effect to occur poses many challenges for physical and psychological domains [41]. To date, research involving art dosage interventions has been mainly carried out in relation to mental well-being. For example, recent meta-analytical work investigating the effect of music listening on reducing anxiety has found that approximately half an hour of music listening had an effect on decreasing anxiety levels [42]. Similarly, Tymoszuk and colleagues [43] found that visiting art museums every few months rather than once a year or never helps reduce loneliness in older adults, suggesting that more frequent art engagement might have a protective effect against loneliness. However, the extent to which different art knowledge training doses impact judgements of artworks and their generalizability effects to novel contexts remains unexplored.

A third key question concerns the cognitive and brain systems that support the development of art knowledge expertise. It seems intuitive that perceptual, cognitive and affective systems could be involved, and emerging evidence shows that studio artists versus non-artists demonstrate better perceptual and attentional flexibility and greater top-down control over visuospatial attentional processing [31,44–47]. Other research, by contrast, has reported no differences between artists and non-artists on visual cognition [48,49]. Therefore, to date, based on a relatively small set of studies, there is some evidence in support of the claim that the expertise of studio artists can have an impact on cognitive function, as well as some work showing no differences.

However, the extent to which cognitive systems associated with executive functions of attention are re-shaped with developing art knowledge expertise remains largely unexplored. Executive functions are a set of abilities required for goal-directed behaviour usually involving mental shifting, information updating and response inhibition [50,51]. One medical research study by Dolev *et al*. [52] investigated whether art knowledge training using representational paintings enhances visuospatial skills in medical students. They found that students participating in an art history course had better scores than controls at describing photographs containing medical disorders, suggesting that art knowledge might enhance visual observational skills. However, the type and structure of cognitive systems that might be associated with the development of art knowledge remain largely unexplored. Indeed, the extent to which a relatively short art training intervention (approx. 20 min) can impact executive resources is as yet unknown. Furthermore, our wider motivation to study the impact of art training on executive resources is partly grounded in scepticism regarding the possibility that art training (or other relatively brief interventions) can robustly impact executive functions. For example, brief mindfulness interventions often claim to be able to impact executive function [53,54]. Before researchers and wider society get carried away on the possibility that art training can similarly impact general cognitive systems, such as executive functions, we wanted to empirically establish the extent to which art training can benefit cognitive function. As such, the motivation to study the impact on executive functions is less theoretically driven and more empirical and practical, in terms of robust detection of phenomena.

Developing a deeper understanding of how art knowledge impacts aesthetic evaluations, as well as the plasticity of cognitive systems, is valuable for several reasons. From a basic science perspective, generalizability is an important but frequently ignored or underappreciated issue in psychology [55]. The extent to which effects of interest generalize across a multitude of situational factors, such as participants, stimuli, laboratories and cultures, among others, deepens understanding of the boundaries that govern where and when learning generalizes to new contexts. In addition, a greater understanding of the cognitive mechanisms involved in plasticity helps to build a more mechanistic understanding of how art experience shapes perception and cognition.

From an applied perspective, understanding dose–response profiles and generalizability are equally important. If art can be useful as an intervention to assist with physical and mental development benefits [26,56], it is crucially important to know the optimal dose for an effect to occur, and the extent to which the effects of art interventions generalize beyond the specifics of the study, such as the setting (e.g. museum or laboratory), or the particular style of painting or dance. Without such knowledge, the impact of art interventions in the real world would be considerably reduced.

The current study aims to deepen our understanding of the impact of art knowledge training on aesthetic judgements and on executive functions that support knowledge acquisition. Across three pre-registered experiments, using a training intervention paradigm and a Bayesian multi-level regression modelling approach to data analysis, we investigate the ways in which (i) art training knowledge impacts subsequent aesthetic judgements of artworks (Experiment 1); (ii) different art knowledge training doses impact subsequent aesthetic judgements of artworks (Experiment 2); and (iii) art versus non-art training knowledge affect executive functions of attention (Experiment 3).

## 2. Experiment 1

### 2.1. Introduction

In Experiment, 1 we investigated two key research questions. First, we examined the extent to which different judgements of artworks are impacted by an art knowledge training session. Second, we examined the extent to which the impact of art knowledge training on judgements of artworks can generalize to previously unseen artworks. We predicted that participants would assign greater ratings of aesthetic preference, aesthetic understanding, affective judgement and aesthetic skill for trained artworks (i.e. those they learn about during the art lesson) than for untrained artworks. Also, we predicted that the impact of the art training on judgements of artworks would generalize to unseen art as a function of how similar the unseen art is to the training materials. Specifically, less similarity between artworks discussed during the art training and unseen art would lead to a reduced impact on judgements.

The hypotheses for Experiment 1 are rooted in the idea that prior knowledge about artworks contributes to art understanding and appreciation. Abundant previous research has demonstrated that contextual art knowledge, such as artworks’ titles or curatorial descriptors, aids meaningful interpretation of artworks and enhances judgements of liking [57–63]. The main explanatory framework is linked to fluency processing theory [64], according to which content-related art knowledge increases the ease (or fluency) of processing and artwork understanding, which leads to greater artwork liking judgements.

Furthermore, the generalization hypothesis is based on the near and far transfer of learning research. Accordingly, near transfer refers to an improved skill in a similar trained skill whereas far transfer refers to an enhanced skill that is different to the trained skill [35,36]. Given that, we reasoned that learning about one particular Impressionist artist’s work would increase subsequent aesthetic judgements about novel, unseen artworks produced by the same Impressionist artist and the impact of training would reduce as the generalization target was further from the training material.

### 2.2. Method

#### 2.2.1. Transparency and openness—pre-registration and open science statement

Across all three experiments, the research questions, hypotheses, planned analyses, sample sizes and exclusion criteria were pre-registered before data collection started. For Experiment 1, the pre-registration can be accessed at https://aspredicted.org/7r5t-jgrg.pdf. In addition, consistent with recent metascience proposals [65], the raw data, stimuli and analysis code for each experiment are available online on the open science framework (https://osf.io/fpmxq/). By doing so, others can pursue tests of alternative hypotheses, as well as more exploratory analyses and meta-analyses.

#### 2.2.2. Ethics statement

All the experimental procedures for Experiment 1 were granted ethical approval by the Research Ethics and Governance Committee of the School of Human and Behavioural Sciences at Bangor University, United Kingdom (Ethics number— 2018-16460-A14807). All participants provided informed consent before completing the experiment. This experiment did not include fieldwork and no other permissions were required.

#### 2.2.3. Participants

All participants across Experiment 1 were recruited from Bangor University’s Psychology student pool system for course credit. In addition, participants were screened for visual art expertise. All participants in Experiment 1 were recruited during November–December 2021. The sample size was determined by the largest participant number we could recruit given the resources available for multiple connected experiments. This approach is consistent with Lakens [66], who emphasized that sample sizes in research are inherently constrained by available resources. Given that, we pre-registered to test 100 participants.

101 participants were recruited in Experiment 1 (85 females, 16 males, Mean_age_ = 20.94, SD_age_ = 6.14, age range = 18 to 49). We found no visual art experts in our experiment sample. The visual art expertise results are reported in electronic supplementary material, figure S1. Participants were excluded if they answered correctly ≤ 3 questions out of 7 questions on our art knowledge post-training questionnaire, which was set to assess the extent to which participants were paying attention during the art training lesson. Therefore, the final sample included 71 participants (55 females, 16 males, Mean_age_ = 21.55, SD_age_ = 6.14, age range = 18 to 49). The results from the follow-up art training questionnaire are reported in electronic supplementary material, figure S2.

#### 2.2.4. Stimuli, design, tasks and procedure

##### 2.2.4.1. Art stimuli

The art images consisted of representational artworks derived from three Western nineteenth and twentieth century art styles: Realism, Impressionism and Post-Impressionism. The current art style categorization is based on art history standards in establishing and evaluating artistic styles, which recognizes distinct visual features among these three art styles (e.g. composition, pictorial space, form, line, colour, light, tone, texture [67–76]).

The Realism artworks consisted of 20 images describing either human bodies (10 images) or landscapes (10 images). The Realism artwork was taken from the art stimuli set used in previous work [77,78]. The Impressionist artworks consisted of 40 images by Spanish artist, Joaquín Sorolla y Bastida (1863−1923), 20 images describing human bodies and 20 landscapes. Out of these 40 images by Sorolla, 20 images were used in both the pre- and post-training, whereas 20 images were only used in the post-training. The Post-Impressionist artworks consisted of 20 images by French artist, Paul Gauguin (1848−1903), 10 images describing human bodies and 10 landscapes. The Impressionist and Post-Impressionist stimuli were obtained from the freely available online visual arts encyclopaedia, WikiArt (https://www.wikiart.org). Each image was sized to be 785 × 774 pixels.

Furthermore, all art stimuli were matched according to several criteria. Regarding the stimuli depicting people, approximately half of the stimuli contained three and fewer than three people, whereas the other half depicted more than three people. Moreover, given the differences in art style, the Realism and Sorolla stimuli depicted Western people, whereas Gauguin depicted a combination of Western (four stimuli) and non-Western people (six stimuli). Concerning the landscape stimuli, approximately half of the stimuli illustrated water scenes (e.g. seascapes, ponds, sea storms), whereas the other half showed landscapes (e.g. gardens, parks, mountains, cities).

**Figure 1.**
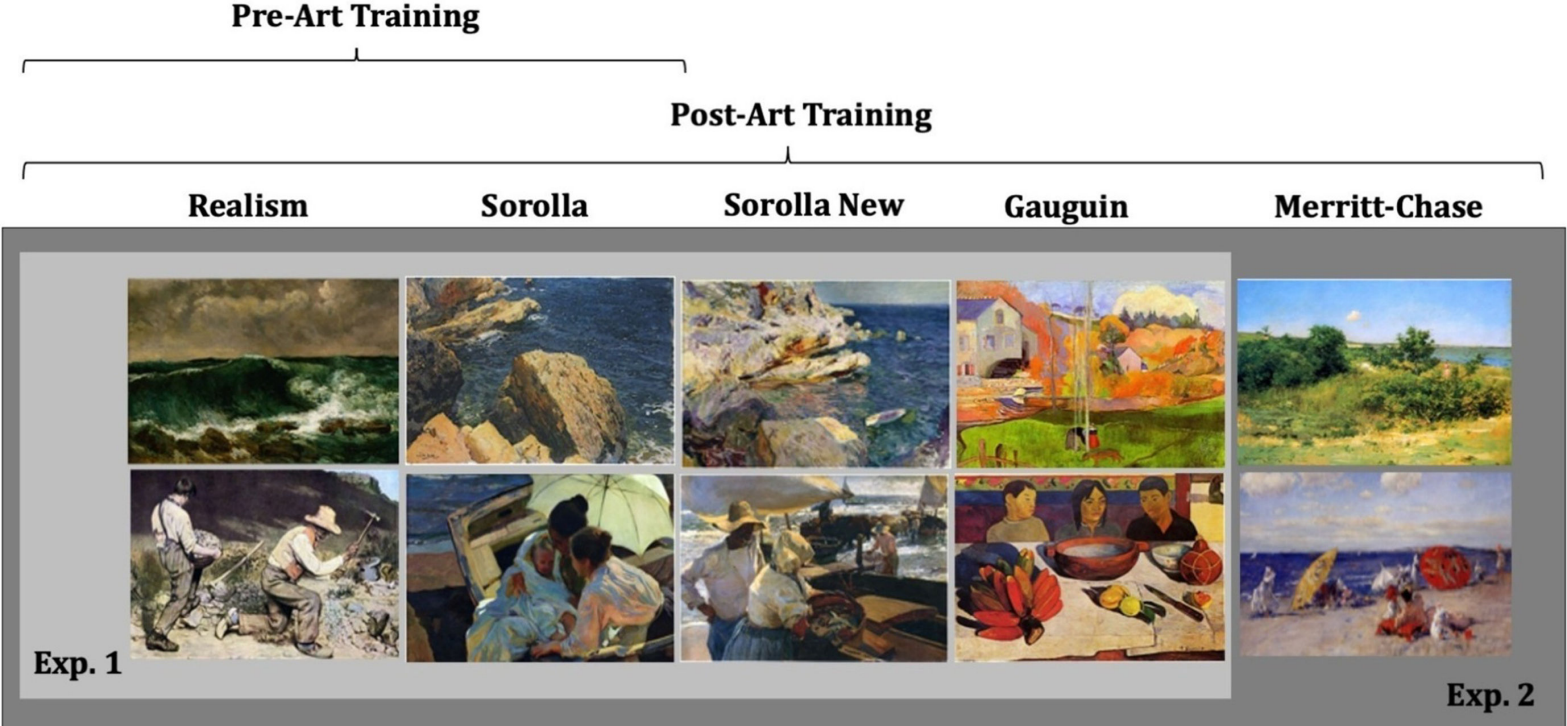
Categories of art stimuli used in Experiments 1 and 2, across pre- and post-art training. In Experiment 1, the pre-art training involved the presentation of 20 Realism stimuli and 20 Impressionist stimuli by Sorolla. The post-art training included the art stimuli from the pre-training (20 Realism stimuli and 20 Impressionist stimuli by Sorolla) and also 20 new art stimuli by Sorolla and 20 new Post-Impressionist art stimuli by Gauguin. Stimuli for Experiment 2 were similar to Experiment 1 with one exception. The post-art training also included 20 art stimuli by Merritt-Chase.

For a complete description of the stimuli used in Experiment 1, including the list of artworks, artists, year of production and museum collection, see electronic supplementary material, table S8. Copyright permitting, all the art stimuli that we used are also available on our open science framework page (https://osf.io/fpmxq/). Sample images from each image category can be seen in figure 1.

##### 2.2.4.2. Art training video

Art knowledge training consisted of watching a 22-minute video that discussed various aspects of pictorial art by Spanish artist Joaquín Sorolla y Bastida. The video comprised an art history lesson on 20 paintings by Sorolla, out of which 10 depicted human bodies and 10 depicted landscapes. The paintings discussed during the art training video were also evaluated by participants during the pre-training and post-training sessions across different dimensions of aesthetic experience. The art training video was created considering the typical competencies and learning objectives of a guided art museum tour, such as explaining, analysing, storytelling and contextualizing artefacts. The aim was to facilitate a rich learning experience that would broaden participants’ awareness of Sorolla’s art and would strengthen their ability to think critically and express interpretations based on what they see [2,79,80].

In conceptualizing and producing the art training content, several aspects were considered, including input from art history and empirical aesthetics. Based on art history research, we first employed an iconographic analysis, as a way of understanding the meaning that artwork had at the time it was created [81–83]. We, therefore, focused on describing the artwork’s subject (pre-iconographic stage), classifying the artwork’s content according to cultural interpretation conventions at the time (iconographic stage), followed by broader interpretations considering the general historical context (iconological stage). Second, we incorporated elements of formal and stylistic analysis to discuss the visual properties of the artworks, such as composition, viewpoint, pictorial space, form, line, colour, light to understand the shared characteristics of Impressionism’s artistic style.

As emphasized by art history research, formal analysis aims to study the visual elements of artworks and to analyse the contribution of such elements to the overall impression of artwork [84–87]. In addition, the stylistic analysis helped to demonstrate that artworks have common visual features among artists working at the same historical time [83,84,88,89], and therefore enabled us to classify the artworks as belonging to Impressionist artistic style. Third, we used biographical interpretation to demonstrate how stories about Sorolla’s personal life can reveal and enrich the meaning of the artwork itself [87,90]. Finally, we used elements of critical theory to inform how societal and political structures have influenced Sorolla’s art and Impressionism, in general, as an artistic period [83,86,87,90].

There were three main reasons for choosing art knowledge training based on Sorolla’s art. First, Sorolla’s art is representational, and we aimed for art training that requires moderate effort from participants to process the information. Previous evidence suggests that representational art is more easily processed and more preferred than abstract art [91–95]. Second, we aimed for an art style that is usually liked by non-experts. In this regard, prior research has demonstrated that art styles, such as Realism or Impressionism, are highly liked by lay people [96–101].

Third, we aimed to introduce an artist whose name or pictorial art is mostly unknown today to the UK public, and therefore to provide participants with a motivating new learning opportunity. Ironically, Sorolla was one the most celebrated artists worldwide during his lifetime, but his popularity diminished after the 1920s. The first Sorolla exhibition in the UK in over a century organized by the National Gallery was held in 2019 [102], highlighting that for more than 100 years, Sorolla was absent from the British museological circuit. On this basis, as well as our participant screening questions, we fully expected Sorolla and his artwork to be unfamiliar to most of our sample.

In terms of learning outcomes, we hoped that by the end of the art training participants would gain an understanding and appreciation of Sorolla’s art. That would include knowledge of formal visual elements (e.g. colour, light, line), aspects of style characteristics (e.g. compositional framing effects, brushstrokes effects), art skill (e.g. trompe l’oeil effects, light rendering), historical value and social relevance that would inform and build aesthetic judgements. To our knowledge, this is one of the first tools employed in the field to comprehensively deliver a visual art knowledge lesson in a non-expert sample. The art knowledge training video is available on our open science framework page (https://osf.io/fpmxq/).

##### 2.2.4.3. Design

**Figure 2.**
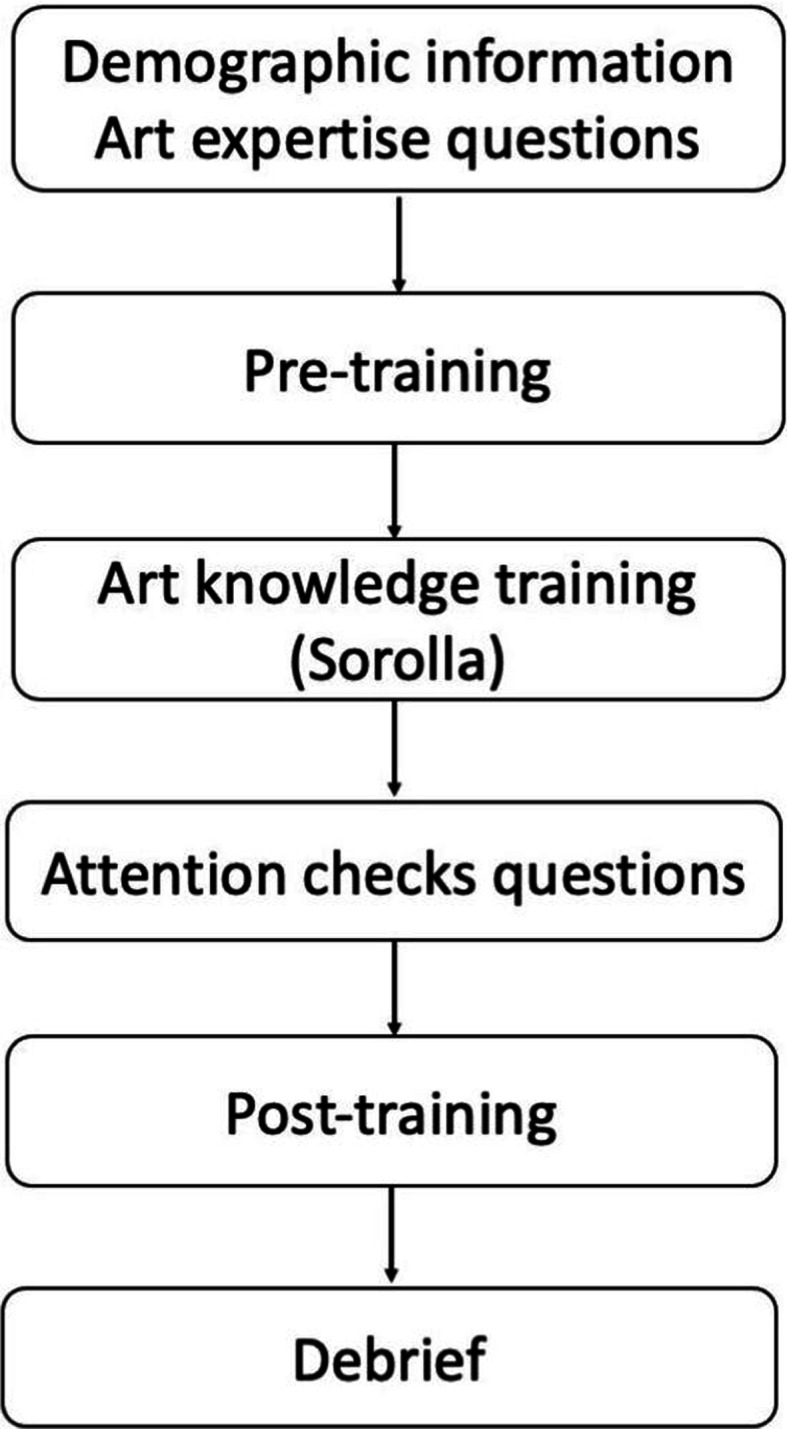
A visual description of the order of the tasks in Experiment 1. Both pre-training and post-training consisted of ratings on aesthetic preference, understanding, affective judgement and artistic skill judgement.

This study used a within-participant design, which comprised pre-training, art knowledge training and post-training (figure 2). The pre-training included two conditions (pre-Realism, pre-Sorolla), whereas the post-training phase included four conditions: pre-Realism, pre-Sorolla, post-Sorolla New and post-Gauguin. More details on these conditions are provided below. The dependent variables were aesthetic preference, aesthetic understanding, affective judgement and artistic skill judgement.

##### 2.4.4.4. Tasks and procedure

Experiment 1 included three main components presented in the same order to all participants: pre-training, art knowledge training and post-training. Both the pre- and post-training consisted of rating tasks, whereas the training comprised an art training video lesson. The experimental tasks were produced in PsyToolkit [103,104]. The completion of this online experiment was restricted to laptop and desktop users only; tablets and mobile phones were not permitted. Participants were instructed to complete the whole experiment in one sitting. The experimental procedure is illustrated in figure 2.

**Figure 3.**
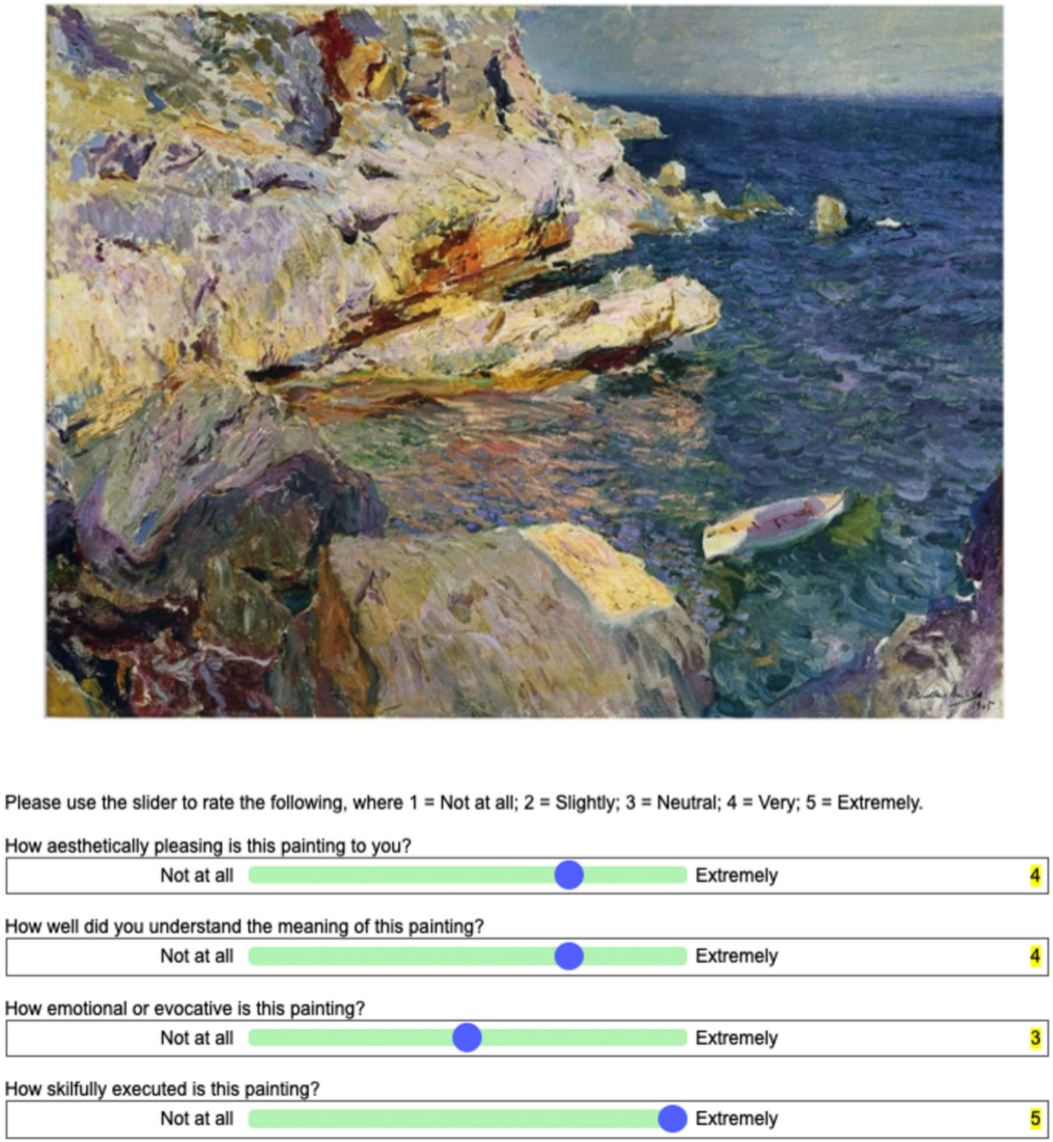
Example of experimental trial during pre- and post-training.

Pre-training involved participants rating 40 paintings (20 Realism art stimuli and 20 art stimuli by Sorolla) on four variables: (i) aesthetic preference (‘how aesthetically pleasing is this painting to you?’); (ii) aesthetic understanding (‘how well did you understand the meaning of this painting?’); (iii) affective judgement (‘how emotional or evocative is this painting?’); (iv) artistic skill judgement (‘how skilfully executed is this painting?’). Therefore, pre-training involved a total of 160 ratings. All ratings were assessed on a 5-point Likert scale (1–5; not at all—extremely). The paintings remained on the screen until participants made a rating response. The order of the paintings was randomized across participants. For a graphical illustration of the task, please see figure 3. To avoid confusion, before the pre-training, each aesthetic judgement was defined, and each rating scale point was explained by using examples.

The training session consisted of an art knowledge video based on Sorolla’s pictorial art, as described in detail previously. Before the art knowledge training, participants were instructed about the following: (i) the art video’s length (22:08 minutes); (ii) to carefully watch the art video as they would have to complete art knowledge follow-up questions; (iii) to wear earphones or to adjust the volume on their laptops or desktop computers to clearly hear the art training narrative; (iv) to watch the art video in its entirety in order to continue the post-training phase.

The content of the video consisted of the same 20 art stimuli that were presented during the pre-training rating task (pre-Sorolla). The order of paintings with people versus landscapes was fixed. The first part of the art video discussed 10 paintings describing people, whereas the second part discussed 10 landscape paintings. To avoid language intelligibility effects [105,106], the content of the art training video was narrated by a native English speaker. After the video art training, participants were asked to complete seven multiple-choice questions (MCQs) to gauge the level of art knowledge acquired during the training lesson.

To ensure that all seven MCQs tested the art knowledge gained through the art training and not the general art knowledge accumulated prior to the art training, we tested the response accuracy of all seven MCQs in a separate pilot before the main experiment started (20 participants, 15 females, 5 males, Mean_age_ = 31.48, SD_age_ = 2.95). This pilot data showed that out of seven MCQs, only one question had 35% response accuracy across all participants. This suggests that it was more of a general knowledge question, and we changed that question in the main experiment to make sure that learning was tied more specifically to the video material. The pilot results are reported in electronic supplementary material, figure S3.

The post-training involved participants rating 80 paintings. Out of 80 paintings, 20 were the same Realism paintings from the pre-training, 20 were the same Sorolla paintings from the pre-training and art video training. Also, there were 20 new paintings by Sorolla that shared similar style features with Sorolla’s paintings from pre-training and video training. Moreover, there were 20 new paintings by Gauguin, characterized by a post-Impressionist art style representation. All 80 paintings were rated on the same four variables as in the pre-training: (i) aesthetic preference; (ii) aesthetic understanding; (iii) affective judgement; (iv) artistic skill judgement. Therefore, there was a total of 320 ratings during the post-training. All other features of the post-training were the same as the pre-training. The whole testing procedure lasted about 70 minutes for most participants (Mean_time_ = 69.66 minutes, SD_time_ = 23.80 minutes).

### 2.2.5. Data analyses

We preregistered a Bayesian estimation approach to multi-level regression modelling [107]. We used two main approaches to evaluate our hypotheses. First and foremost, we reported and discussed the posterior distribution of our key parameters of interest within the most complex model. The most complex model had the maximum number of varying parameters that the design permitted (Barr et al., 2013). From the most complex model, we described the posterior (density) distribution of key parameters and noted the median point of the distribution as well as the lower and upper bounds of the 95% quantile intervals. These intervals were then used to make inferential judgements regarding our pre-registered hypotheses. As such, since we are not using null hypothesis significance testing, we will not be reporting *p*-values and we will not be making statements about statistical significance.

More practically, we used a recent translation of McElreath’s [107] general principles into a different set of tools (Kurz, 2020), which use the Bayesian modelling package ‘brms’ to build multi-level models (Bürkner, 2017, 2018). Moreover, our data wrangling approach follows the ‘tidyverse’ principles (Wickham & Grolemund, 2016) and we generate plots using the associated data plotting package ‘ggplot2’, as well as the ‘tidybayes’ package (Kay, 2020). All of these analytical approaches were performed in the R programming language [108].

Given that the dependent variables are an ordered category (a 1−5 rating scale), we used ordinal regression. We ran two different types of ordinal regression model—one for each question of interest. The first multivariate model included all four DVs and addressed the extent to which different judgements of artworks are impacted by art knowledge training. The second multivariate model included all four DVs and addressed the extent to which the impact of the art training on judgements of artworks generalizes to previously unseen artworks.

For the first research question—pre- versus post-art training effects—we calculated nine multivariate models, which were built incrementally in complexity (note—the models were multivariate in the sense that they included multiple dependent measures). We first computed an intercepts-only model bpp0.1, then we added varying thresholds (model bpp0.2), just so that we could compare subsequent models that included predictors of interest to models without any predictors. Model bpp0.3 included varying item intercepts, whereas model bpp0.4 included varying participant intercepts. Next, we added predictors for training (bpp1), and image type (bpp2). Then, we added the interaction between training and image type (bpp3). Finally, we added correlated varying intercepts for items (bpp3.2) and correlated varying intercepts and effects for participants (bpp3.3). Model bpp3.3 was the full model. For all of these models, we allowed thresholds to vary by item. The formula for the full model (model bpp3.3) is specified below:


$$\begin{array}{l} \text{ brms formula = bf(mvbind(preference, understanding, affect, skill) | thres(4, gr=item)}\\ \;\;\;\;\;\;\;\;\;\;\;\;\text{ \textasciitilde{} 1 + training * image\_type +}\\ \;\;\;\;\;\;\;\;\;\;\;\;\;\text{(1 |p| item) +}\\ \;\;\;\;\;\;\;\;\;\;\;\;\text{ (1 + training * image\_type |a| participant))} \end{array}$$

Note: training = pre- versus post-art training; image_type = Realism versus Sorolla; item = stimulus number.

Factors were coded according to a deviation coding style, where factors sum to zero and the intercept can then be interpreted as the grand mean and the main effects can be interpreted similarly to a conventional anova. As such, both training and image type were coded as −0.5 (pre/Realism) and 0.5 (post/Sorolla).

For the second research question, we estimated generalization effects. We calculated seven models, which were built incrementally in complexity. We first computed an intercepts-only model bg0.1, then we added varying thresholds (model bg0.2). Model bg0.3 included varying item intercepts, whereas model bg0.4 included varying participant intercepts. Next, we added condition (pre_Sorolla, post_Sorolla, post_Sorolla_new, post_Gauguin) as a factor (bg1). Then, we added the correlated varying intercepts and effects for participants (bg1.2), and the correlated varying effects for item intercepts (bg1.3). Model bg1.3 was the full model. The formula for the full model (model bg1.3) is given below:


$$\begin{array}{l} \text{ brms formula = bf(mvbind(preference, understanding, affect, skill) | thres(4, gr=item)}\\ \;\;\;\;\;\;\;\;\;\;\;\;\text{ \textasciitilde{} 1 + condition +}\\ \;\;\;\;\;\;\;\;\;\;\;\;\;\text{(1 |p| item) +}\\ \;\;\;\;\;\;\;\;\;\;\;\;\text{ (1 + condition |a| participant))} \end{array}$$

We set priors using a weakly informative approach [109]. The priors used in Experiment 1 are provided in electronic supplementary material, table S1. Weakly informative priors are distinct from uniform priors by placing a constrained distribution on expected results rather than leaving all results to be equally likely (i.e. uniform). Weakly informative priors are also distinct from specific informative priors, which are more precisely specified because we currently do not have sufficient knowledge to place more specific constraints on what we expect to find. Considering the relatively small effects in psychology in general, we placed priors for the thresholds (or intercepts) at zero with a normal distribution of 1.5. The fixed effects or predictors, as well as the standard deviations, were centred around zero with a normal distribution of 1. Also, by using weakly informative priors, we allow for the possibility of large effects, should they exist in the data [109–112]. A further advantage of weakly informative priors is that we would not expect the choice of prior, as long as it remained only weakly informative, to matter too much because the data would dominate the structure of the posterior distribution.

A few specific parameters were of particular interest in evaluating our key hypotheses. Given that all participants undertook the art knowledge training about Sorolla’s art we first expected an overall effect of training (post > pre) on aesthetic judgements of artworks and an interaction term showing that the training effect is larger for Sorolla than Realism paintings. Also, since the art stimuli by Sorolla were explained in depth during the art training and rated again during the post-training (post_Sorolla), we expected an effect of condition. Specifically, we expected post_Sorolla to show the largest training effect followed by post_Sorolla_new (unseen art stimuli by Sorolla) and then post_Gauguin (unseen art stimuli by Gauguin). Effects that would show substantial overlap with zero would suggest training has had minimal to no impact on judgements.

### 2.3. Results

The models’ chains were carefully monitored, and the convergence diagnostics did not raise any concerns. The chains can be seen in electronic supplementary material, figure S8A,B. For further details regarding the number of iterations and chains, please see our analysis code on the open science framework (https://osf.io/fpmxq/).

#### 2.3.1. Pre- and post-training effects

**Figure 4.**
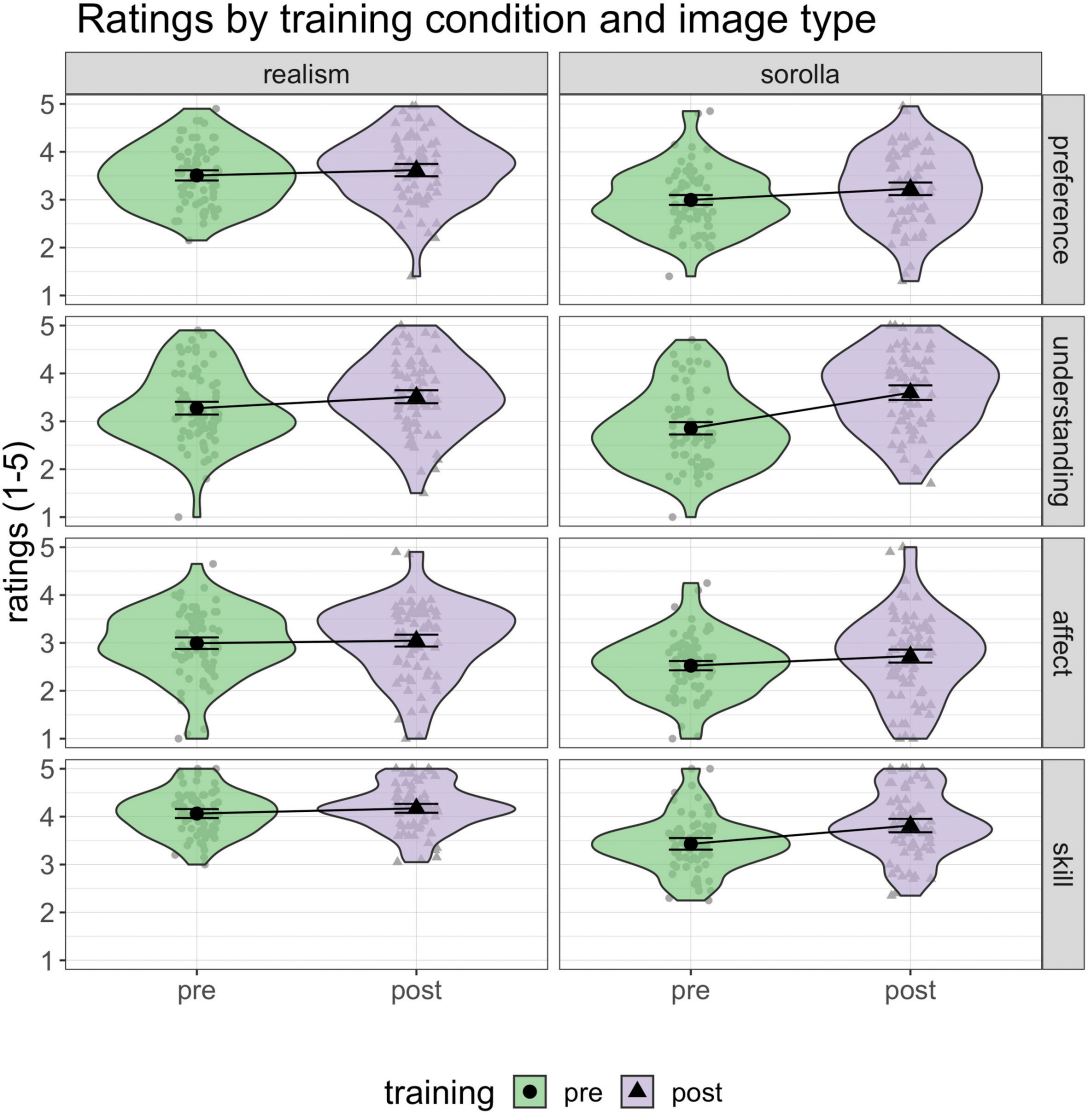
Ratings across pre- and post-art training on Realism and Sorolla images for all four DVs. The left panel shows the pre- and post-training ratings for Realism, whereas the right panel shows the pre- and post-training ratings for Sorolla. The four rows illustrate our main DVs—from the top row for aesthetic preference, then understanding, to the bottom rows that show the ratings for affect and artistic skill. The ratings are reported on a 5-point Likert scale (1 = not at all to 5 = extremely). Error bars represent 95% confidence intervals. The black markers (circles and triangles) and interval estimates represent the group mean average, whereas the grey markers represent the individual participants.

Rating summary data for all four dependent variables (preference, understanding, affect, artistic skill) across pre- and post-training conditions and for Realism and Sorolla images are shown below (figure 4).

**Figure 5.**
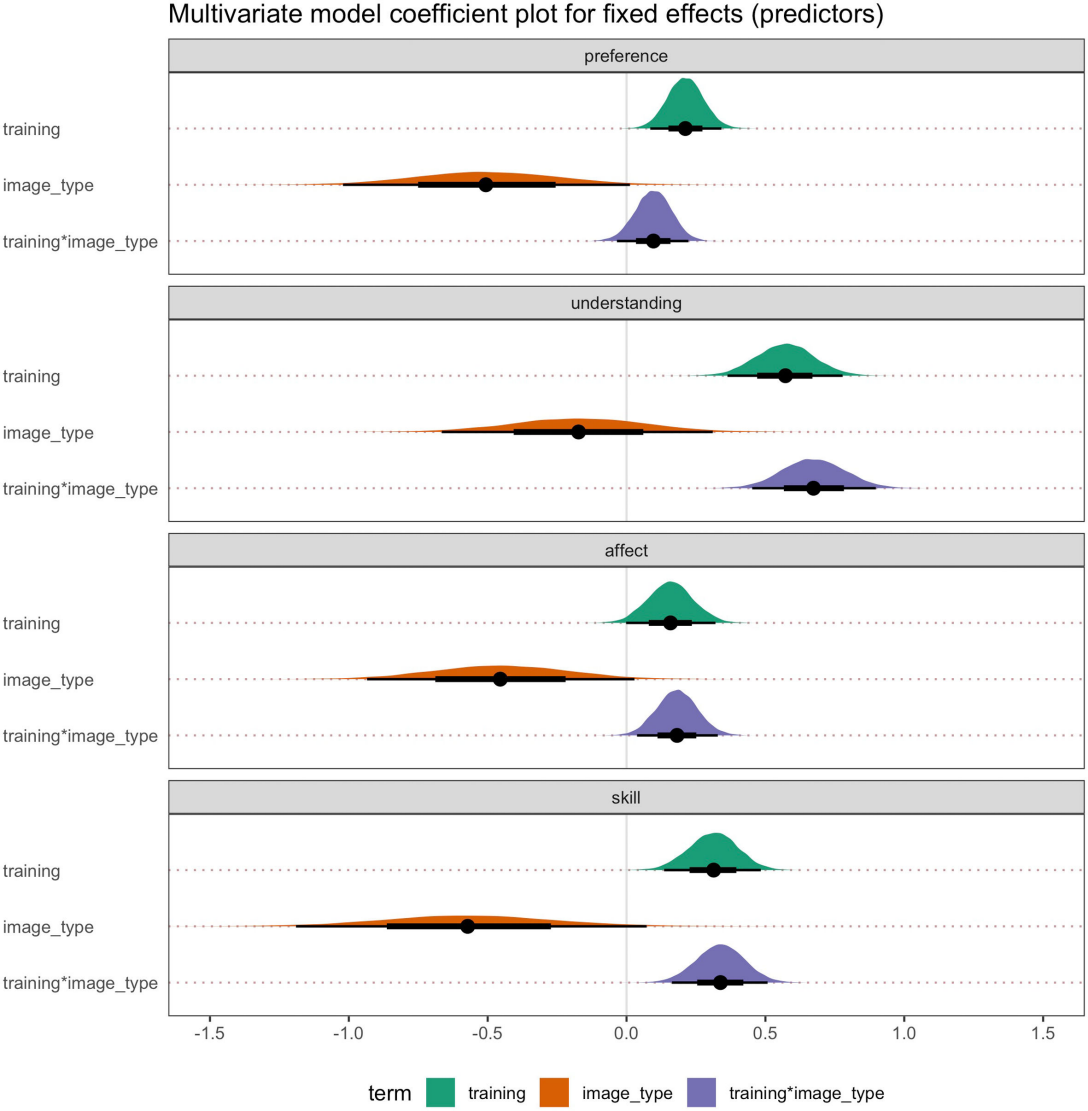
Multivariate parameter estimates for the full model (Model 9) across all four dependent variables: preference, understanding, affect and artistic skill. *Note:* training = pre versus post; image_type = image category (Realism versus Sorolla); training*image_type = interaction between training (pre versus post) and image_type (Realism versus Sorolla). Point estimate = median; error bars represent 66% quantile intervals (thick black lines) and 95% quantile intervals (thin black lines).

Parameter estimates for the most complex model (bpp3.3) across all four dependent variables are shown in figure 5 and electronic supplementary material, table S3. The posterior distribution for the main predictors across all dependent variables indicated a largely positive response for the effect of training (post > pre) and for the interaction between training and image type, whereas image type (Sorolla > Realism) was negative. The training*image_type interaction illustrates that the training effects are larger for Sorolla paintings than for Realism paintings, which can also be seen visually in the raw data plots (figure 4). Therefore, these results provide support for the effectiveness of the art knowledge training on judgements of artworks for trained rather than untrained artworks. In other words, there was a greater effect on ratings of Sorolla than Realism at post-training compared with pre-training.

We also performed a model comparison analysis, with results detailed in the electronic supplementary material, figure S6.

#### 2.3.2. Generalization effects

**Figure 6.**
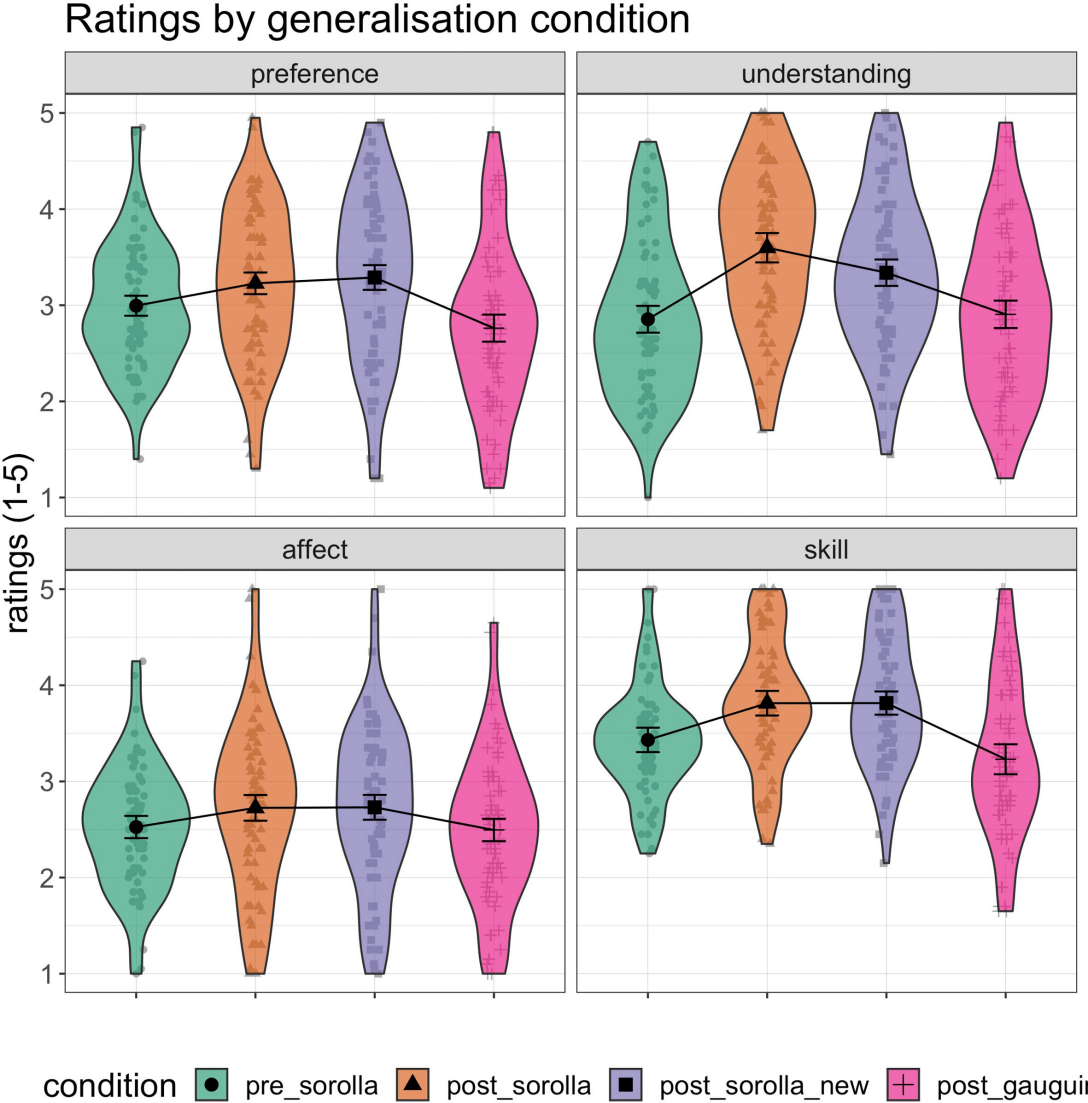
Ratings across generalization conditions (pre-Sorolla compared with post-Sorolla, post-Sorolla new and post-Gauguin) for all four DVs. The top panels show the generalization ratings for aesthetic preference (left) and understanding (right), whereas the bottom panels show the ratings for affect (left) and artistic skill (right). The ratings are reported on a 5-point Likert scale (1 = not at all to 5 = extremely). Error bars represent 95% confidence intervals. The black markers (circles, triangles, squares, crosses) and interval estimates represent the group mean average, whereas the grey markers represent the individual participants.

Rating summary data for all four dependent variables (preference, understanding, affect, artistic skill) across generalization conditions are shown below (figure 6).

**Figure 7.**
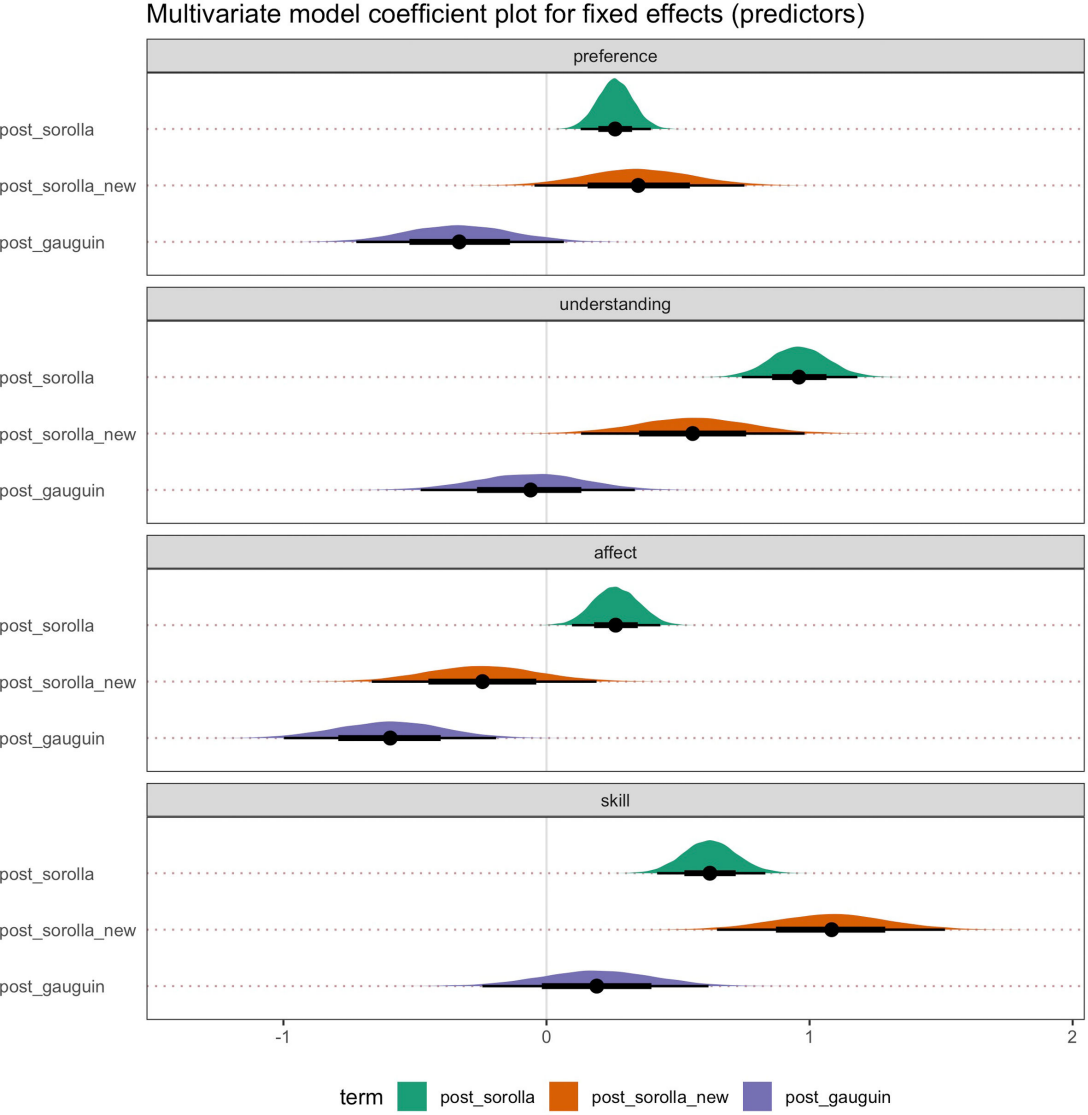
Multivariate parameter estimates for the full model (Model 7) across all four dependent variables: preference, understanding, affect and artistic skill. Note: post_Sorolla = artworks by Sorolla presented during post-training; post_Sorolla_new = previously unseen artworks by Sorolla presented during post-training; post_Gauguin = previously unseen artworks by Gauguin presented during post-training. Point estimate = median; error bars represent 66% quantile intervals (thick black lines) and 95% quantile intervals (thin black lines).

Parameter estimates for the most complex model (Model 7) are shown in figure 7 and electronic supplementary material, table S4. The posterior distribution for the main predictors indicated a largely positive response for post_Sorolla followed by post_Sorolla_new and post_Gauguin on understanding, artistic skill, affective and preference judgements. These results show that compared with pre_Sorolla, the judgements of artworks were greater for post_Sorolla artworks. In addition, the judgements of artworks generalized to previously unseen artworks by Sorolla rather than unseen Gauguin’s artworks, suggesting that generalization effects depend on how similar the unseen artworks are to the trained artworks.

We also performed a model comparison analysis, with results detailed in the electronic supplementary material, figure S7.

### 2.4. Discussion

Experiment 1 showed that art knowledge training led to greater ratings on understanding, artistic skill, aesthetic preference and affective judgement for trained rather than untrained artworks. Therefore, we support our first hypothesis regarding pre- versus post-training effects and show that 22 minutes of an art knowledge training video session increases art understanding and art appreciation [33,34]. Experiment 1 also demonstrated that the impact of the art training on judgements of artworks generalizes to previously unseen artworks as a function of how similar the unseen art is to the training materials. Specifically, the greater similarity between artworks discussed during the art training and unseen art determined a greater impact on judgements.

## 3. Experiment 2

### 3.1. Introduction

In Experiment 2, we examined two key research questions. First, we investigated the extent to which various judgements of artworks are impacted by different art knowledge training doses. Second, we examined to what extent different doses of art training impact the ability of training to generalize to previously unseen artworks. We reasoned that by comparing four art knowledge training doses, we may observe differential effects on judgements of artworks and on generalizability effects to novel artworks that would help determine the optimal art training dose required to impact aesthetic judgements. From a basic research perspective, investigating dose–response effects is important because it deepens our understanding of the plasticity of cognitive systems that support learning in this context. In addition, from an applied perspective, knowing which doses lead to robust effects on judgements could guide the time-efficient and effective design of future interventions in art museums or schools. Previous research from educational learning has indicated that across different learning contents and learners’ characteristics, a lesson length of approximately 30−45 minutes is optimal for retention of the trained material [113], suggesting that learning is linked to session duration.

### 3.2. Method

#### 3.2.1. Pre-registration

We used the same design and analysis pipeline as in Experiment 1, all of which we pre-registered in advance of the experiment commencing. The pre-registration file for Experiment 2 can be found at https://aspredicted.org/rtx8-mqv7.pdf. We note a minor deviation from the pre-registered analysis regarding model comparison. Given that the current study followed up the main results from Experiment 1 with four different training doses, we aimed to evaluate the key parameters in the full model, rather than assess model comparison across all incremental models, as this is computationally expensive and not essential for our primary inferences.

#### 3.2.2. Ethics statement

All the experimental procedures for Experiment 2 were granted ethical approval by the Research Ethics at Macquarie University’s School of Psychological Sciences, Sydney, Australia (Ethics number—13177). All participants provided informed consent before completing the experiment. This experiment did not include fieldwork and no other permissions were required.

#### 3.2.3. Participants

All participants in Experiment 2 were recruited from Macquarie University as part of a class tutorial. Consistent with the approach used in Experiment 1, the sample size was determined based on resource availability. As such, we aimed to collect as many participants as possible from the class tutorial. Students attending the tutorial were not compelled to take part in the study if they did not wish to. They were provided with an alternative task to complete, such as reading a relevant journal article, with no repercussions for participants’ academic performance. Participants completed this experiment either in person in a classroom setting (*n* = 657) or online (27). All participants in Experiment 2 were recruited in August 2022.

Participants were randomly recruited to one of the four experimental groups. 188 participants (141 females, 46 males, 1 unspecified, Mean_age_ = 21.96, SD_age_ = 6.53, age range = 18 to 57) were recruited for the first dose art training group. 173 participants (122 females, 50 males, 1 unspecified, Mean_age_ = 21.66, SD_age_ = 4.63, age range = 18 to 52) were recruited for the second dose art training group. 145 participants (104 females, 39 males, 2 unspecified, Mean_age_ = 21.72, SD_age_ = 5.91, age range = 18 to 55) were recruited for the third dose art training group. 178 participants (127 females, 50 males, 1 unspecified, Mean_age_ = 21.38, SD_age_ = 5.05, age range = 18 to 62) were recruited for the fourth dose art training group.

The exclusion criteria were identical to Experiment 1. Participants were excluded if they answered ≤ 3 questions out of 7 questions on our post-art knowledge follow-up questionnaire, which assessed the extent to which participants were paying attention during the art training lesson. Therefore, the final sample for the first dose included 132 participants (96 females, 35 males, 1 unspecified, Mean_age_ = 22.12, SD_age_ = 6.93, age range = 18 to 57). The final sample for the second dose consisted of 112 participants (77 females, 35 males, Mean_age_ = 22.27, SD_age_ = 5.11, age range = 19 to 52). The final sample for the third dose consisted of 111 participants (81 females, 29 males, 1 unspecified, Mean_age_ = 21.68, SD_age_ = 4.99, age range = 19 to 49). The final sample for the fourth dose included 125 participants (92 females, 32 males, 1 unspecified, Mean_age_ = 21.76, SD_age_ = 5.61, age range = 19 to 62).

The results for post-training followed-up questions for all experimental groups are reported in electronic supplementary material, figure S12. We also report the visual art expertise results in electronic supplementary material, figure S11.

#### 3.2.4. Stimuli, design, tasks and procedure

##### 3.2.4.1.Art stimuli

The art images were identical to those used in Experiment 1, with one main exception. In addition to Realism paintings, Sorolla’s Impressionist artwork, and Gauguin’s Post-Impressionist artwork, we introduced new Impressionist artwork by the American artist, William Merritt-Chase (1849−1916). The main rationale for this new experimental condition was that Merritt-Chase’s pictorial art is described as Impressionist in artistic style [114], and therefore similar to Sorolla’s art. Having a new experimental condition that would share stylistic feature similarity (e.g. line, shape, form, colour, space, texture) with the training materials would help test the hypothesis that judgements of artworks would generalize to unseen artwork as a function of how similar the style of unseen artwork is to the art training materials.

Merritt-Chase’s artwork matched the same criteria of Experiment 1, specifically, that there were 20 images in total, 10 images describing human bodies and 10 landscapes. Also, the art stimuli by Merritt-Chase were obtained from freely available online visual arts encyclopaedia, WikiArt (https://www.wikiart.org). Each image was sized to be 785 × 774 pixels. For a complete description of the stimuli used in Experiment 2, please see the electronic supplementary material. Copyright permitting, all the art stimuli that we used are also available on our open science framework page (https://osf.io/fpmxq/). Sample images from each image category can be seen in figure 1.

##### 3.2.4.2.Art training videos

The art knowledge training content was similar to Experiment 1; however, it was split into four doses. The first art training dose was 5:03 minutes and contained an art history lesson that referenced four paintings by Sorolla (two human bodies and two landscapes). The second dose was an art history lesson of 10:18 minutes, which depicted eight paintings by Sorolla (four human bodies and four landscapes). The third dose was 16:04 minutes and consisted of an art history lesson that referenced 16 paintings by Sorolla (eight human bodies and eight landscapes). The fourth was the full training (22:08 minutes), which was identical to Experiment 1 and contained an art history lesson on 20 paintings by Sorolla (10 human bodies and 10 landscapes). The paintings discussed during the art training video were also evaluated by participants during the pre-training and post-training sessions across different dimensions of aesthetic experience.

##### 3.2.4.3.Design

**Figure 8.**
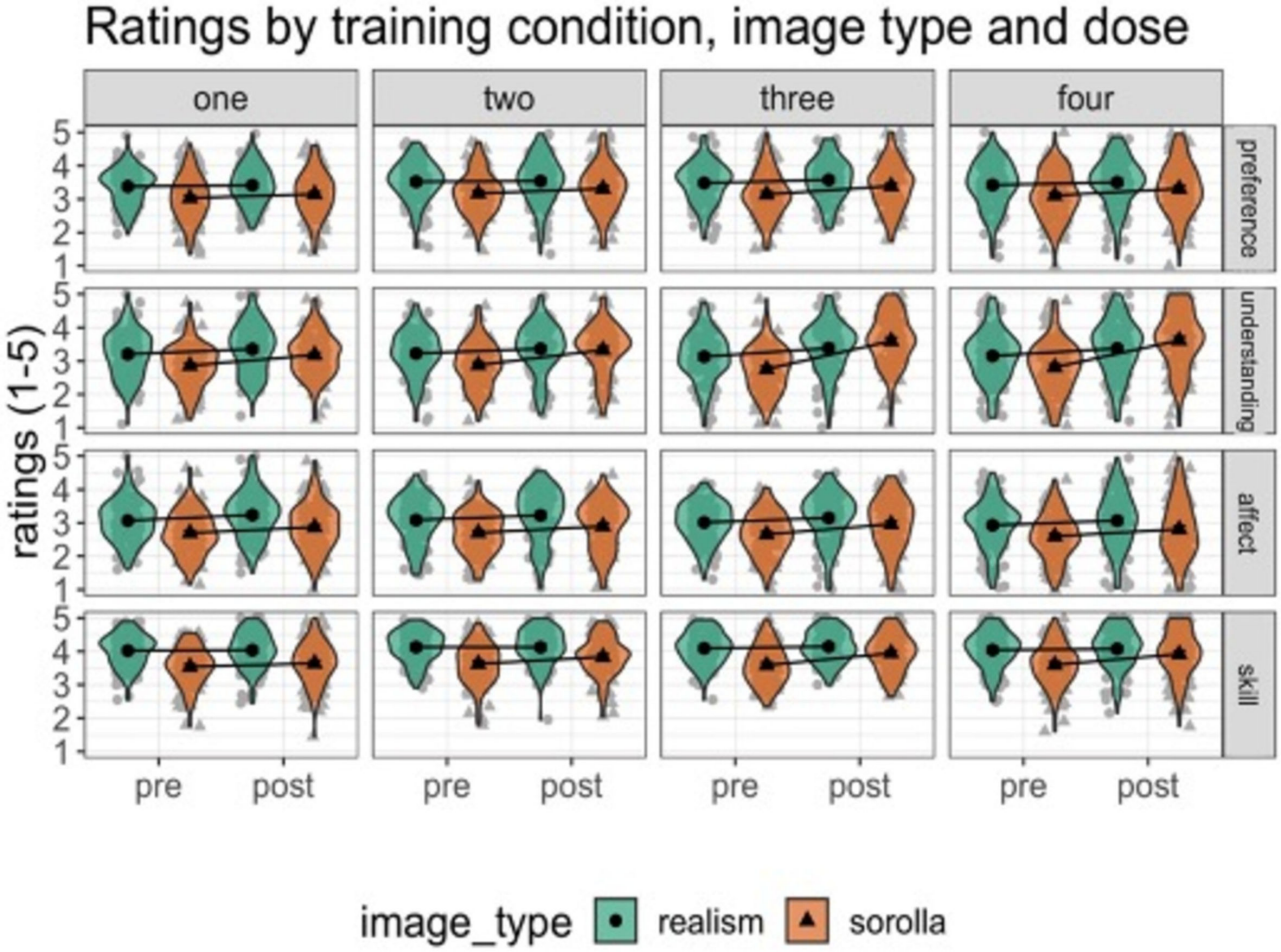
Ratings across training (pre- versus post-), image type (Realism versus Sorolla) and dose (one, two, three, four) on all four DVs. The columns show all four art training doses, whereas the rows show the ratings for aesthetic preference, understanding, affect and artistic skill. The ratings are reported on a 5-point Likert scale (1 = not at all to 5 = extremely). Error bars represent 95% confidence intervals. The black markers (circles and triangles) and interval estimates represent the group mean average, whereas the grey markers represent the individual participants.

This study used a mixed within- and between-participant design, which comprised pre-training, art knowledge training and post-training (figure 8). All participants completed pre-training and post-training sessions, which included aesthetics ratings of artworks in a within-participant design (2 × session: pre and post). A further within-participant design contained seven conditions: pre-Realism, pre-Sorolla, post-Realism, post-Sorolla, post-Sorolla New, post-Gauguin, post-Merritt-Chase. Participants were also randomly assigned to one of four art training doses in a between-participant manipulation (4 × dose: dose 1 art training video 5:03 minutes, dose 2 art training video 10:18 minutes, dose 3 art training video 16:04 minutes, dose 4 art training video 22:08 minutes). The dependent variables were the same as in Experiment 1.

##### 3.2.4.4.Tasks and procedure

**Figure 9.**
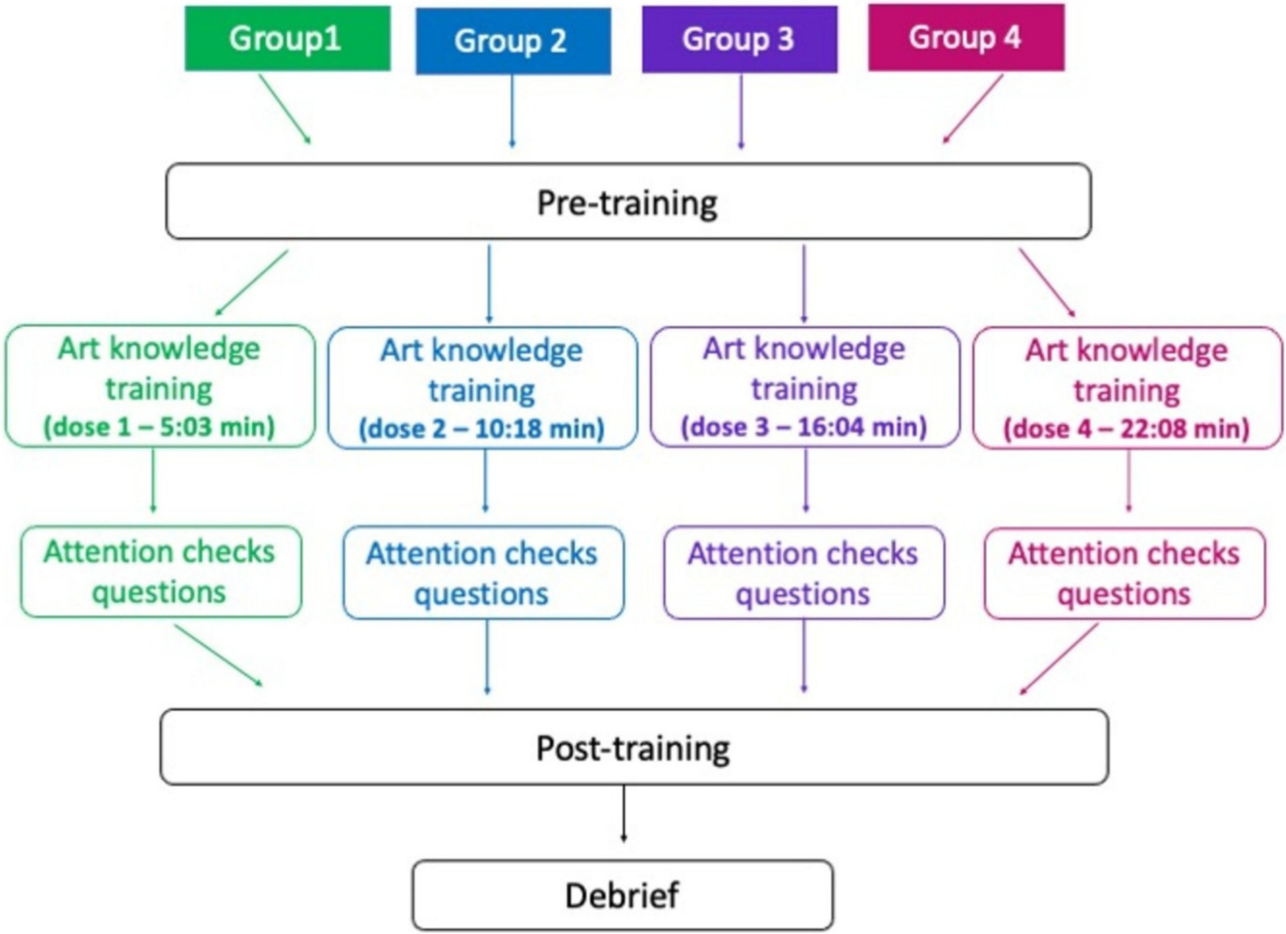
A visual description of the order of the tasks in Experiment 2. Both pre-training and post-training consisted of ratings on aesthetic preference, understanding, affective judgement and artistic skill judgement.

All the experimental tasks and procedures were similar to Experiment 1, with two exceptions. First, there were four doses of the art knowledge training. Second, we introduced a new post-training condition in which participants would rate Impressionist paintings by Merritt-Chase. The experimental procedure is illustrated in figure 9.

Therefore, the post-training involved participants rating 100 paintings. Out of 100 paintings, 20 were the same Realism paintings from the pre-training session, and 20 were the same Sorolla paintings from the pre-training session. Also, there were 20 new paintings by Sorolla that shared similar style features with Sorolla’s paintings from pre-training. In addition, there were 20 new paintings by Gauguin and 20 new paintings by Merritt-Chase. All 100 paintings were rated on the same four variables as in the pre-training: (i) aesthetic preference; (ii) aesthetic understanding; (iii) affective judgement; (iv) artistic skill judgement. Therefore, there was a total of 400 ratings during the post-test.

### 3.2.5. Data analyses

We used the same general approach to data analyses as performed in Experiment 1. For the first research question—pre- versus post-art training effects as a function of art training dose—the full model was similar to Experiment 1, except for including the art training dose condition. Model bpp5.3 was the full model. The formula for the full model (model bpp5.3) is specified below:


$$\begin{array}{l} \text{ brms formula = bf(mvbind(preference, understanding, affect, skill) | thres(4, gr=item)}\\ \;\;\;\;\;\;\;\;\;\;\;\;\text{ \textasciitilde{} 1 + training * image\_type * dose +}\\ \;\;\;\;\;\;\;\;\;\;\;\;\;\text{(1 |p| item) +}\\ \;\;\;\;\;\;\;\;\;\;\;\;\text{ (1 + training * image\_type |a| participant))} \end{array}$$

*Note:* training = pre versus post-art training; image_type = Realism versus Sorolla; dose = art training dose one (5:03 minutes), dose two (10:18 minutes), dose three (16:04 minutes), dose four (22:08 minutes); item = stimulus number.

For the second research question, we estimated generalization effects similar to Experiment 1, depending on condition and dose. Model bg3.3 was the full model.

The formula for the full model (model bg3.3) is specified below:


$$\begin{array}{l} brms\;formula=bf(mvbind\;(preference,understanding,affect,skill)|thres\;(4,gr=item)\\ \;\;\;\;\;1+condition*dose+\\ \;\;\;\;\;(1|p|item)+\\ \;\;\;\;(1+condition|a|participant)) \end{array}$$

*Note:* condition = pre_Sorolla, post_Sorolla, post_Sorolla_new, post_Merritt_Chase, post_Gauguin; dose = art training dose one (5:03 minutes), dose two (10:18 minutes), dose three (16:04 minutes), dose four (22:08 minutes); item = stimulus number.

As in Experiment 1, we had a few parameters that were of particular interest in assessing our key hypotheses, as follows:

1) Since all four groups of participants undertook a different art training dose about Sorolla’s art, we first expected an overall effect of training (post>pre) on aesthetic judgements of artworks and an interaction term showing that the training effect is larger for Sorolla than Realism paintings, and also larger for the greater art training doses.

2 a) Given that the art stimuli by Sorolla were explained in depth during the four art training doses and rated again during the post-training (post_Sorolla), we expected the post_Sorolla condition to have the largest effect, followed by post_Sorolla_new (unseen art stimuli by Sorolla), post_Merritt_Chase (unseen art stimuli by Merritt-Chase, similar stylistically to Sorolla), and then post_Gauguin (unseen art stimuli by Gauguin, dissimilar stylistically to Sorolla).

2b) Since we used four different art training doses, we expected an interaction showing that the condition effect is largest for post_Sorolla in the greater art training doses than for pre_Sorolla.

3) Given the lack of previous research in relation to art training dosage, there was no clear expectation about the lowest art training dose required to produce an effect on judgements of artworks.

Effects that would show substantial overlap with zero would suggest training has had minimal to no impact on judgements.

### 3.3. Results

The models’ chains were carefully monitored, and the convergence diagnostics did not raise any concerns. The chains can be seen in electronic supplementary material, figure S17.

#### 3.3.1. Pre- and post-training effects

Rating summary data for all four dependent variables (preference, understanding, affect, artistic skill) across pre- and post-training conditions (pre-Realism, pre-Sorolla, post-Sorolla_new, post-Merrit-Chase, post-Gauguin) and as a function of the art training doses (one—5:03 minutes; two—10:18 minutes; three—16:04 minutes; four—22:08 minutes) are shown below (figure 8).

**Figure 10.**
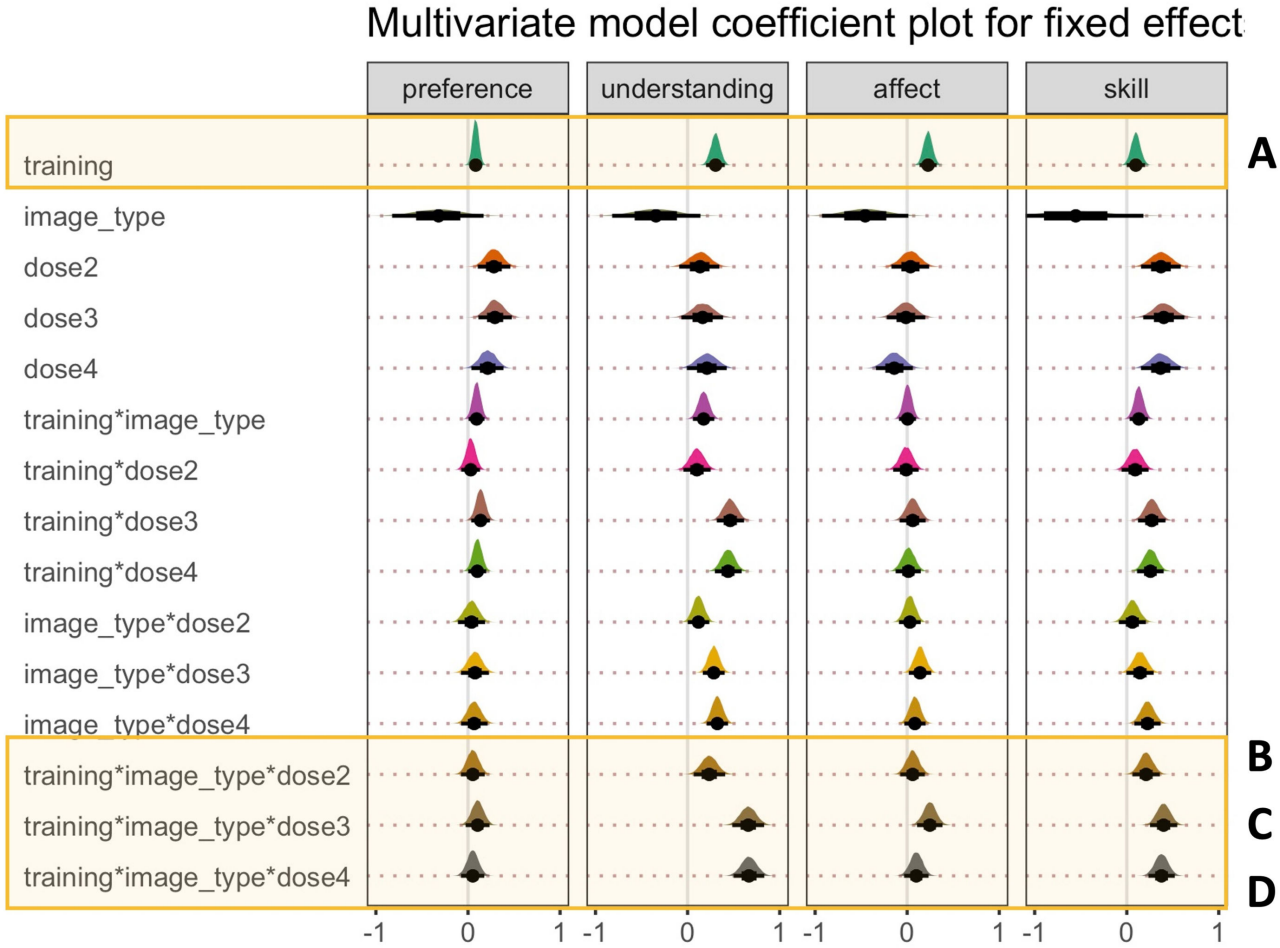
Multivariate parameter estimates for the full model (Model 13) across all four dependent variables: preference, understanding, affect and artistic skill. The highlighted panels show the main parameters of interest. Panel (A) = average effect of training; Panels (B*–*D) = three-way interaction between training, image type and dose. *Note:* training = pre versus post; image_type = image category (Realism versus Sorolla); dose = one—5:03 minutes; two—10:18 minutes; three—16:04 minutes; four—22:08 minutes. Point estimate = median; error bars represent 66% quantile intervals (thick black lines) and 95% quantile intervals (thin black lines).

Parameter estimates for the most complex multivariate model (bpp5.3) are shown in figure 10 and electronic supplementary material, table S5. While we visualize the full model, we only discuss the main parameters of interest (please see the highlighted panels in figure 10). First, we consider the three-way interactions (panels (*b*–*d*)). For the understanding and skill DVs, the posterior distribution for the three-way interactions between training, image type, and dose illustrated a positive response. The interaction terms were also numerically larger for dose 3 and 4 than for dose 2. Here, a positive value response means that the training effect was larger for Sorolla than Realism paintings and for larger doses compared with the smallest dose (dose 1). Therefore, these results suggest that participants assigned greater judgements of artworks for understanding and artistic skill, for Sorolla rather than Realism and after watching longer art training videos (16:04 and 22:08 minutes) rather than shorter art training videos (10:18 minutes). At least in terms of judgements of understanding and skill, these findings highlight the effectiveness of the art knowledge training on judgements of artworks for trained rather than untrained artworks. Moreover, these results suggest that the success of art knowledge training is more effective for the two larger doses (3 and 4) than smaller doses.

By contrast, for the preference and affect DVs, the three-way interaction terms showed much more overlap with zero, which is consistent with no clear three-way interactions between training, image type and dose. Instead, for the preference, understanding and skill DVs, there were positive interactions between training and doses 3 and 4, which shows that the general effects of training were larger at doses 3 and 4 than dose 1.

Next, we consider the average effect of training (panel *a*). While all the judgements showed a positive response, the judgements of affect and understanding illustrated a larger numerical response than preference and artistic skill judgments. A positive value response for the average effect of training means greater judgements for post-art training rather than for pre-art training.

#### 3.3.2. Generalization effects

**Figure 11.**
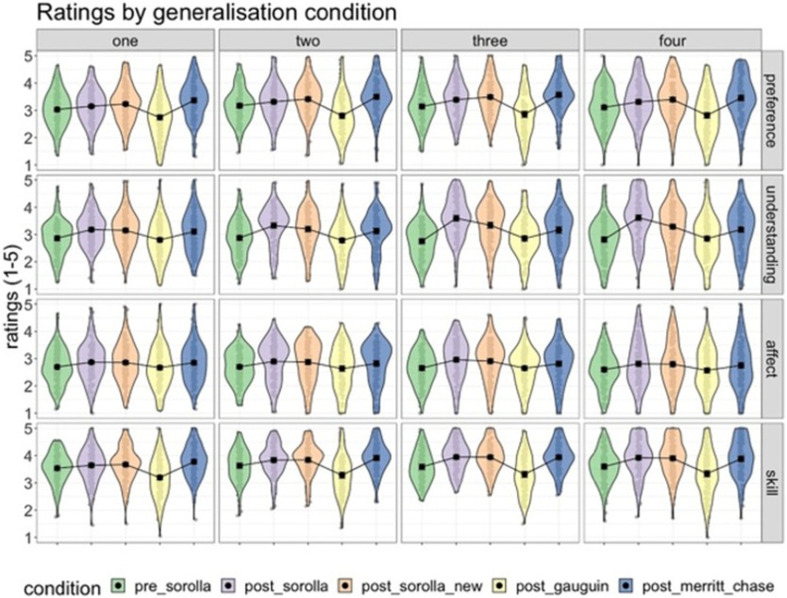
Ratings across dose type and generalization conditions (pre-Sorolla compared with post-Sorolla, post-Sorolla new, post-Gauguin and post-Merritt-Chase) on all four DVs (aesthetic preference, understanding, affect and artistic skill). The columns illustrate the art training doses. The rows show the ratings for aesthetic preference, understanding, affect and artistic skill. The ratings are reported on a 5-point Likert scale (1 = not at all to 5 = extremely). Error bars represent 95% confidence intervals. The black and interval estimates represent the group mean average, whereas the grey markers represent the individual participants.

Rating summary data for all four dependent variables (preference, understanding, affect, artistic skill) across all four art training doses and generalization conditions are shown below (figure 11).

**Figure 12.**
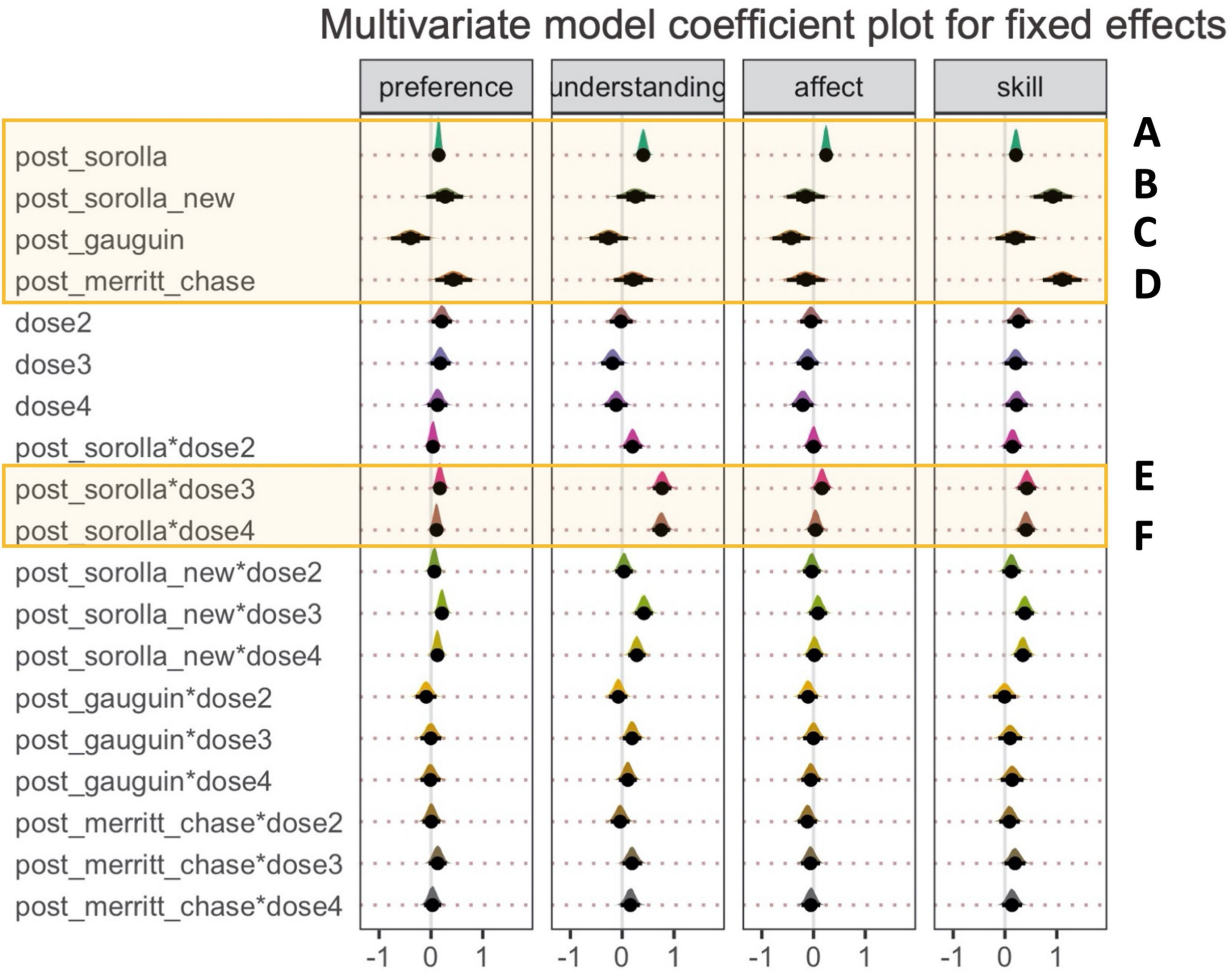
Multivariate parameter estimates for the full model (Model 9) across all four dependent variables: preference, understanding, affect and artistic skill. The highlighted panels show the main parameters of interest. Panels (A–D) = average effect of condition. Panels (E) and (F) = two way interaction between post_sorolla condition and dose. *Note:* post_Sorolla = artworks by Sorolla presented during post-training; post_Sorolla_new = previously unseen artworks by Sorolla presented during post-training; post_Gauguin = previously unseen artworks by Gauguin presented during post-training; post_Merritt_Chase = previously unseen artworks by Merritt-chase presented during post-training; dose = one—5:03 minutes; two—10:18 minutes; three—16:04 minutes; four—22:08 minutes. Point estimate = median; error bars represent 66% quantile intervals (thick black lines) and 95% quantile intervals (thin black lines).

Parameter estimates for the most complex model (bg3.3) across all four dependent variables are shown in figure 12 and electronic supplementary material, table S6. While we visualize the full model, we only discuss the main parameters of interest (please see the highlighted panels in figure 12). First, we consider the two-way interactions in the model (panels (*e*) and (*f*)). For the understanding and skill DVs, the posterior distribution for the interaction between post_Sorolla condition and larger doses (dose 3 and dose 4) showed greater positive effects than pre-Sorolla and dose 2. The interaction terms were also numerically larger for doses 3 and 4 than for dose 2. In this sense, a positive value response means that the condition effect was larger for post-Sorolla than pre-Sorolla and for larger doses than smaller doses.

However, for the preference and affect DVs, the two-way interaction terms showed much more overlap with zero, which is consistent with no clear three-way interactions between post-Sorolla condition and dose. Furthermore, for the understanding and skill DVs, the posterior distribution for the interaction between post_Sorolla_new and larger doses (dose 3 and dose 4) illustrated a positive effect. This means that the condition effect was larger for post-Sorolla_new than pre-Sorolla and for larger doses than smaller doses. Finally, for the affect and preference DVs, there were no clear and consistent interaction effects.

Furthermore, for the average condition effects, the judgements of affect and understanding showed a positive response for the average effect of post_Sorolla condition (panel (*a*)). This means greater judgements for post_Sorolla artworks rather than for pre-Sorolla artworks. In addition, for the skill, preference, and understanding DVs the posterior distribution showed a positive response for the average effect of post_Sorolla_new (panel (*b*)) and Merritt_Chase (panel (*d*)) conditions. This means greater judgements for post_Sorolla_new artworks and Merritt_Chase artworks rather than for pre-Sorolla artworks. However, for the affect, preference, and understanding DVs, the posterior distribution showed a negative response for the average effect of post_Gauguin condition (panel (*c*)), meaning reduced judgements of artworks for post_Gauguin compared with pre-Sorolla.

These results indicate that the judgements of artworks were greater for Sorolla in post-training than the pre-training and also greater for larger training doses than smaller doses. Also, the judgements of artworks generalized to unseen artworks by Sorolla, followed by unseen artworks by Merritt-Chase, and involved larger training doses rather than smaller doses. This suggests that effective generalization effects depend on the artist’s similarity, artistic style similarity and larger training doses.

### 3.4. Discussion

Experiment 2 demonstrated that art knowledge training resulted in greater judgements of understanding and artistic skill for trained artworks (Sorolla) rather than untrained artworks (Realism), and for longer art training videos rather than shorter art training videos. Therefore, in line with our first hypothesis, we show that approximately 22 minutes and approximately 16 minutes of art knowledge training led to stronger effects on judgements for trained materials followed by approximately 10 minutes of training compared with approximately 5 minutes of training.

Furthermore, the effects of art knowledge training generalized to unseen artworks by Sorolla, followed by unseen artworks by Merritt-Chase as a function of larger training doses (approx. 16 minutes and approx. 22 minutes). This highlights that the generalization of judgements of artworks depends on the artist’s similarity and artistic style similarity between the artworks discussed during the art lesson and unseen art, and on longer art training doses. Overall, Experiment 2 shines new light on the generalization effects of training to novel contexts depending on art style similarity and art training session duration.

## 4. Experiment 3

### 4.1. Introduction

Experiment 3 investigated the extent to which executive functions of attention, such as alerting, orienting and executive control, are impacted by art knowledge training. Since visual art expertise has been associated with domain-general executive control [31,44–47], we reasoned that an art knowledge training session would confer greater benefit to reaction time measures of executive functions, such as alerting, orienting and executive control compared with a non-art knowledge training session.

A vast amount of prior research has studied the cognitive and brain systems that support attention and this research has shown that attentional resources are subserved by many different features and dimensions. Here we were interested in three components of attention—alerting, orienting and executive control—as proposed by Posner & Petersen [115]. We chose to focus on these three building blocks of attention for two reasons. First, we wanted to study general aspects of attention that are likely to be applicable in a wide range of settings. Second, in an intervention setting, we felt it was important to choose robust measures, which have been shown to be replicable in past research. We felt that these three measures satisfied these criteria [116] and detail each one in the method section. Furthermore, our motivation to conduct Experiment 3 is partly grounded in scepticism regarding the possibility that art training (or other relatively brief interventions) can robustly impact executive functions. For example, brief mindfulness interventions often claim to be able to impact executive function [53,54]. Before researchers and wider society get carried away on the possibility that art training can similarly impact general cognitive systems, such as executive functions, we wanted to empirically establish the extent to which art training can benefit cognitive function.

Compared with Experiment 1 and 2, we also decided to use a different research design. To gauge the extent to which art training impacts executive functions of attention, we included a comparison non-art training group, which consisted of a video lesson about horticulture and specifically tomatoes. Therefore, in Experiment 3, we used two distinct training groups: an art training group (identical to Experiment 1), and a new, non-art training group. The precise content of the non-art training is explained in detail below.

### 4.2. Method

#### 4.2.1. Pre-registration

We used the same general analysis pipeline as in Experiments 1 and 2, all of which we pre-registered in advance of the experiment commencing. The pre-registration file for Experiment 3 can be found at https://aspredicted.org/4v7g-6ntd.pdf.

#### 4.2.2. Ethics statement

All the experimental procedures for Experiment 3 were granted ethical approval by the Research Ethics and Governance Committee of the School of Human and Behavioural Sciences at Bangor University, United Kingdom (Ethics number—2018-16460-A14807). All participants provided informed consent before completing the experiment. This experiment did not include fieldwork and no other permissions were required.

#### 4.2.3. Participants

In the same manner as Experiment 1, we aimed for a total participant sample of *n* = 100. The sample size was determined considering the same resource availability criteria as in Experiment 1. Participants were randomly recruited for each of the two experimental groups. 50 participants (36 females, 11 males, 3 unspecified, Mean_age_ = 22.40, SD_age_ = 6.90, age range = 18 to 50) were recruited for the art knowledge group. The non-art knowledge group also consisted of 50 participants (42 females, 7 males, 1 unspecified, Mean_age_ = 21.06, SD_age_ = 4.67, age range = 18 to 43). We report the visual art and horticulture expertise results in electronic supplementary material, figures S21. All participants in Experiment 3 were recruited during February–March 2022.

Like Experiments 1 and 2, participants were excluded if they answered correctly ≤ 3 questions out of 7 questions on our post-art knowledge or non-art knowledge follow-up questionnaire, which assessed the extent to which participants were paying attention during the art or non-art training lesson. Therefore, for the art knowledge group, the final sample included 41 participants (33 females, 6 males, 2 unspecified, Mean_age_ = 23.17, SD_age_ = 7.35, age range = 18 to 50). The final sample for the non-art knowledge group consisted of 40 participants (33 females, 6 males, 1 unspecified, Mean_age_ = 21.43, SD_age_ = 5.09, age range = 18 to 43). The results for follow-up questions for both art and non-art groups are reported in electronic supplementary material, figure S22A,B.

#### 4.2.4. Stimuli, design, tasks and procedure

*Art training video*. The art knowledge training was the same as in Experiment 1.

*Non-art training video*. The non-art knowledge training consisted of a 20:17-minute video about tomato crop development and production. The video was carefully matched to the art training video so that it comprised a similar number of words, duration, graphics and narration style. However, the content was different, as the main aim was to create a non-art condition as a benchmark to accurately measure the impact of art knowledge training on executive functions of attention.

The non-art training video content was created considering horticulture science, especially the main findings and directions within tomato crop production [117–120]. Based on previous tomato crop production research, the tomato training video comprised elements of historical background and production techniques. The tomato training video addressed the basic elements of a tomato’s anatomy and the main characteristics of major types of tomatoes. Moreover, other topics were discussed, such as developmental and crop growth processes, chemical composition, visual and sensory factors that contribute to tomato quality, and the most common tomato bacterial diseases. Finally, nascent research highlighting the possible health benefits of tomato consumption was acknowledged [121,122]. Like the art training video, the non-art knowledge video training video is freely available on our open science framework page (https://osf.io/fpmxq/).

The main reason for choosing horticulture knowledge as a control intervention is because the topic or the information presented does not involve art, aesthetics or hedonic-related information. Despite the relative familiarity with tomatoes in everyday life, knowledge about tomatoes from a horticulture science perspective is likely to be scarce, especially in an undergraduate Psychology sample. We screened for horticulture expertise, and we did not find any participants with a particularly deep or wide knowledge of horticulture (or tomatoes) in our sample. In addition, to make sure that the control group was well matched to the art training group, we assured random participation to one of two groups, a similar training video duration, and the same demographic characteristics between the art and non-art groups.

*Design*. Experiment 3 used a within- and between-participant design. Participants completed the Attention Network Test (ANT [123]) pre- and post-training in a within-participant design (2 × session: pre and post). Participants were also randomly assigned to one of two groups in a between-participant manipulation (2 × group: art knowledge versus non-art knowledge). There were three primary dependent variables (alerting, orienting and executive control), which are reaction time measures obtained via the ANT. Alerting scores were calculated as the difference between double cue trials and no cue trials. Orienting was calculated as the difference between spatial cue trials and central cue trials, whereas executive control was gauged as the difference between the congruent target trials and incongruent target trials.

**Figure 13.**
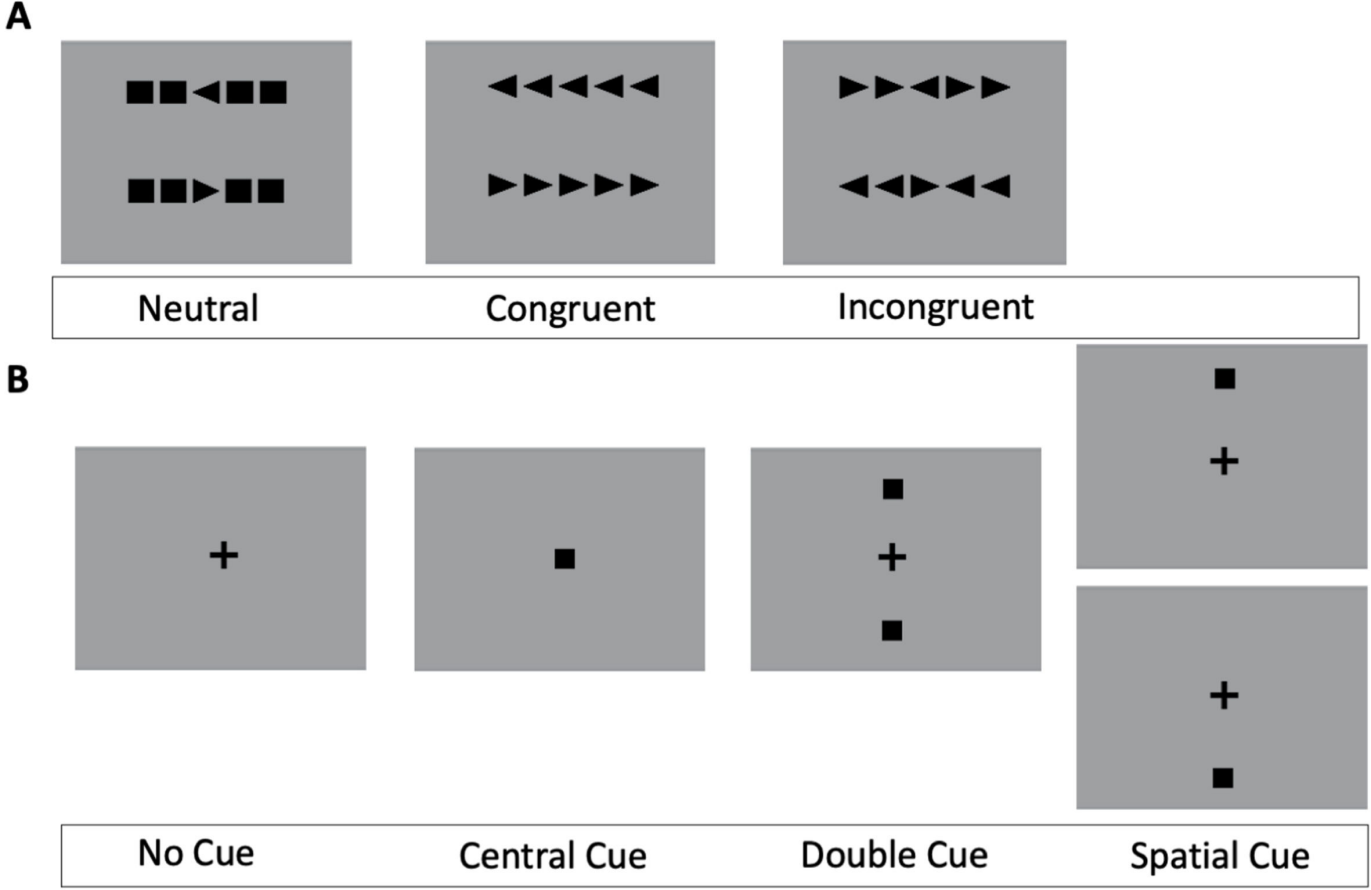
Example of Attentional Network Test (ANT) experimental conditions. Upper panel (A) shows the three flanker types: neutral, congruent, incongruent; lower panel (B) shows the four cue types: no cue, central cue, double cue, spatial cue.

*Tasks and procedure*. Experiment 3 comprised three main components presented in a fixed order: pre-training, art knowledge training or non-art knowledge training and post-training. The main task completed by all participants during the pre- and post-training was the classic version of the ANT (figure 13). Prior to pre-registration and data collection, we conducted a separate behavioural ANT validation experiment (*n* = 13; 2 males, *Mean*_age_ = 20.78, SD_age_ = 3.54). The main aim of this pilot experiment was to ensure that the ANT task worked properly. For full results of the ANT validation experiment, see the electronic supplementary material, figure S25.

The ANT paradigm integrates Posner’s cued reaction time task [124] and Eriksen’s flanker task [125] to separate different attention components. Alerting involves both mental and physical preparation for a fast reaction to stimuli in the environment [115,126]. Orienting prioritizes sensory input by selecting the modality or space location [116]. Executive attention consists of conflict resolution and control in problem-solving, decision-making, error detection and response inhibition [127]. The efficacy and independency of alerting, orienting and executive control have been measured by the Attentional Network Test (ANT) created by Fan *et al*. [123].

The ANT involved the presentation of cues (in the form of one or two squares), which could be used to predict an upcoming target presentation and/or be informative of the target’s location. The square cue phase of the trial was followed by the presentation of target arrows, which could appear individually or in an array of five arrows. Participants were asked to respond as quickly and accurately as possible by indicating whether the central arrow points to the left or right. The central arrow could be congruent or incongruent with the arrows that flank it in the array. The ANT had 12 conditions in total, which spanned 4 cue types (no cue, central, double, spatial) and 3 flanker types (congruent, incongruent, neutral). Each ANT experimental session consisted of one block of 24 practice trials and three blocks of 96 experimental trials. During the practice trials, participants received feedback on their accuracy. An example of ANT experimental conditions is provided in figure 13.

The ANT in both pre- and post-training conditions was produced in PsychoPy (v2020.2.3, Peirce et al., 2019) and run online using Pavlovia. The recruitment was via the Bangor University SONA system. The experiment was restricted to computer and laptop users only. Comprehensive instructions and explanations were given to participants about each part of the study, expectations and requirements. For example, participants were informed about the duration of the study, and were required to complete the experiment in one sitting, on a desk, in front of a computer or laptop, in a well-lit and quiet room. The participants were advised to take part in the study when they were rested and alert.

**Figure 14.**
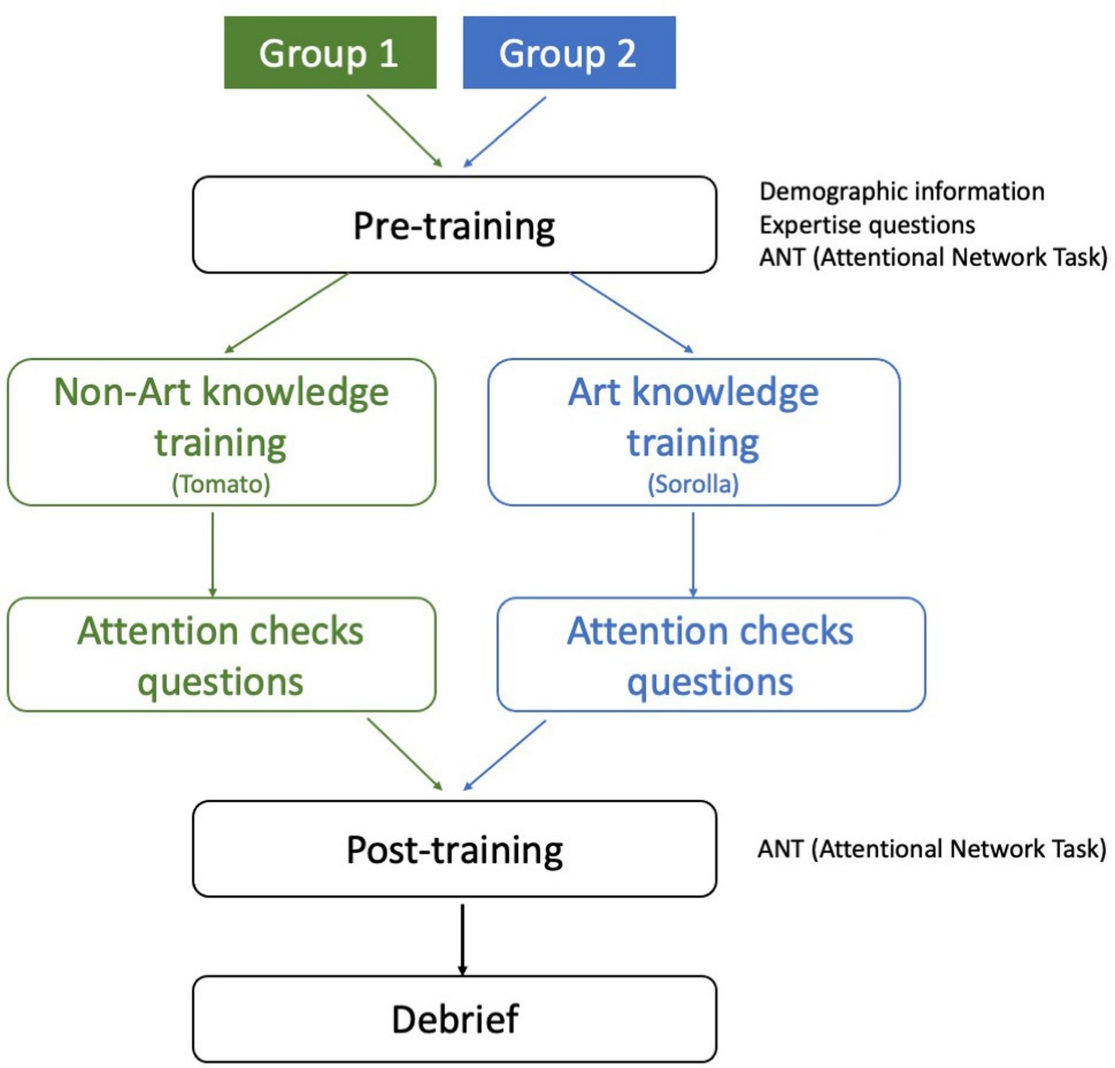
A visual description of the order of the tasks in Experiment 3.

The procedure in Experiment 3 is illustrated in figure 14. Participants were randomly assigned to either the art group or the non-art group. After completing demographic information and expertise questions, all participants undertook the pre-training session, which included the ANT task. Next, participants completed either the art knowledge training where they learned about Sorolla’s pictorial art or the non-art knowledge training where they learned about tomato crop production. After the training phase, participants completed seven follow-up questions to assess participants’ engagement with the training content. Lastly, all participants completed the post-training session, which involved completion of the ANT. Overall, the experiment did not take longer than 60−70 minutes.

As in Experiment 1, to ensure that all seven multiple-choice questions (MCQs) tested the tomato knowledge gained through the tomato training and not the general tomato knowledge acquired prior to the tomato training, we tested the response accuracy of all seven MCQs in a separate pilot before the main experiment data collection started (19 participants, 16 females, 3 males. Mean_age_ = 31.48, SD_age_ = 2.95). None of the seven MCQs had more than 20% response accuracy across all participants, suggesting that the questions were tomato training specific and not easily answered based on general knowledge alone. The pilot results are reported in electronic supplementary material, figure S23.

#### 4.2.5. Data analyses

We used the same general approach to data analyses as performed in Experiments 1 and 2 with one main exception. The primary dependent variables were reaction time measures and we modelled reaction time data using a shifted log-normal regression model, which has previously been shown to be a particularly suitable way to model reaction times (Haines et al., 2020).

We computed 17 models, which were built incrementally in complexity. We first computed an intercepts-only model (b0.1), plus varying intercepts for participants (b0.2), and non-decision time (ndt) parameter per participant (b0.3). Model b1.1 included adding alerting as a predictor of interest, model b1.2 included orienting, and model b1.3 included executive control. We then added predictors for session—pre- versus post (b2.0), followed by two-way interactions between session*alerting (b2.1), session*orienting (b2.2), session*executive (b2.3). Model b3.0 included training group—art versus non-art. We then added two-way interactions between group*alerting (b3.1), group*orienting (b3.2), group*executive (b3.3). Finally, we modelled three-way interactions between session*group*alerting (b4.1), session*group*orienting (b4.2). Model b4.3 was the full model, which additionally included the three-way interactions between session*group*executive. The formula for the full model (model b4.3) is given below:


$$\begin{array}{l} \text{ brms formula = bf(rt \textasciitilde{} 1 + session * group * alerting +}\\ \;\;\;\;\;\text{session * group * orienting +}\\ \;\;\;\;\text{ session * group * executive +}\\ \;\;\;\;\text{ (1 + session * alerting + session * orienting + session * executive | pid),}\\ \;\;\;\;\text{ndt \textasciitilde{} (1 | pid)} \end{array}$$

Note: rt = reaction time in milliseconds; session = pre- versus post-training; group = art versus non art; pid = participant unique identifier; ntd = non-decision time.

Factors were coded according to a deviation coding style. As such, for alerting, orienting and executive, each trial was coded as −0.5 (double cue/spatial cue/congruent), 0.5 (no cue/central cue/incongruent) or 0 (double cue/spatial/congruent). In addition, session and group were coded −0.5 (pre/art) and 0.5 (post/non-art). Similar to Experiments 1 and 2, we set priors using a weakly informative approach [109]. The priors used in Experiment 3 are provided in electronic supplementary material, table S2.

To investigate our key hypotheses, we evaluate the interaction terms in the model to assess the extent to which executive functions of attention vary as a function of session and training group. More specifically, to support our hypothesis, we would expect art knowledge training to lead to greater post-training improvements in reaction time measures than non-art knowledge training. For example, faster reaction time responses to alerting, orienting or executive control during post-art training would represent improvements in attentional functioning.

### 4.3. Results

**Figure 15.**
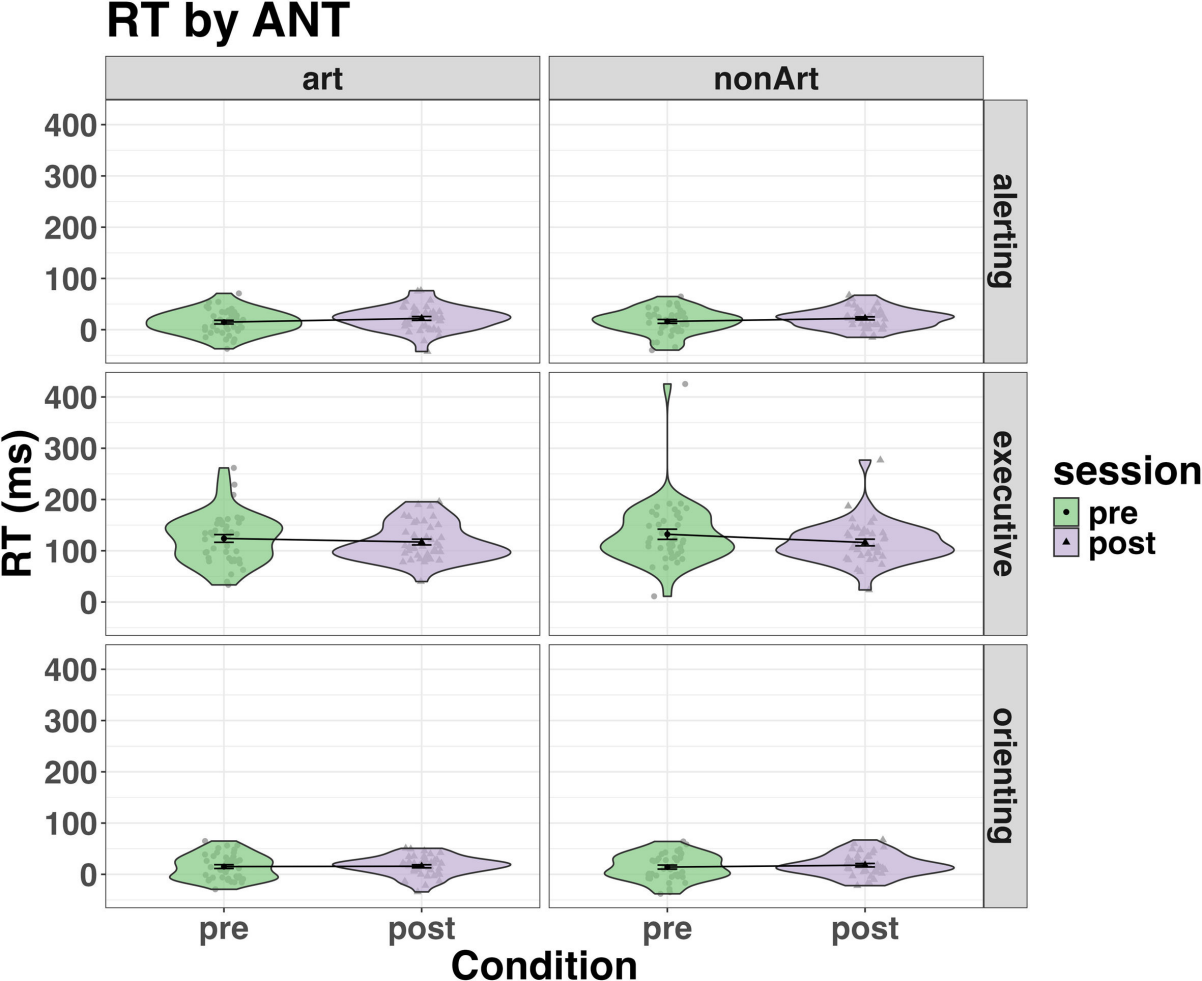
Violin plots on summary data showing ANT reaction time across all conditions. Reaction time is reported in milliseconds (ms). The columns show response times on art versus non-art across pre- and post-training, whereas the rows show the DVs (alerting, executive, orienting). Error bars represent 95% confidence intervals. The black markers (circles and triangles) and interval estimates represent the group mean average, whereas the grey markers (circles and triangles) represent the individual participants.

ANT summary reaction time data for alerting, orienting and executive control across all conditions are shown in figure 15.

**Figure 16.**
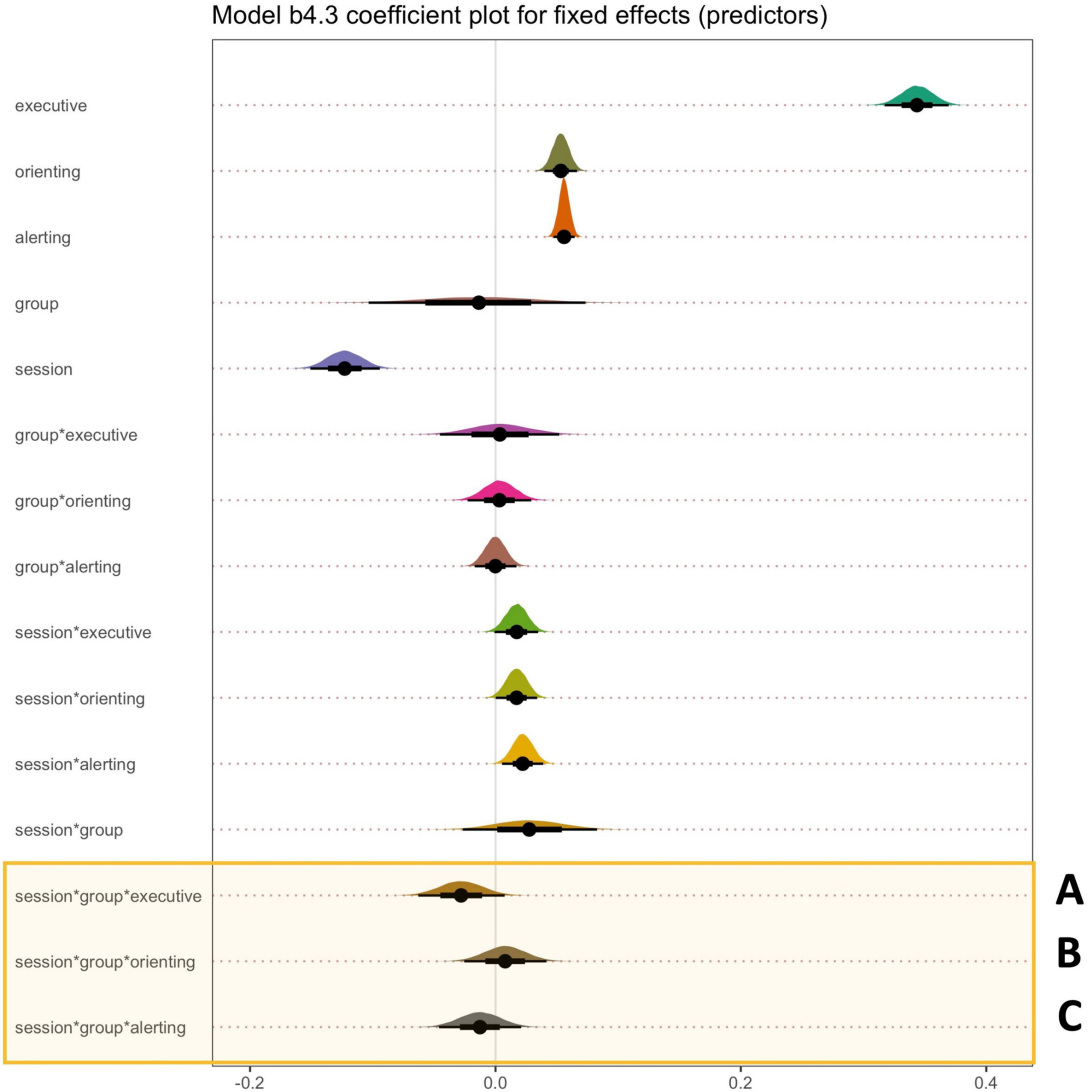
Parameter estimates for each predictor within the full model (Model 17). The highlighted panels show the main parameters of interest. Panels (A–C) = three way interaction effects of session, group and ANT. *Note:* group (art versus non-art); session (pre- versus post-training). Point estimate = median; error bars represent 66% quantile intervals (thick black lines) and 95% quantile intervals (thin black lines).

The parameter estimates for the most complex model (b4.3) across all conditions are shown in figure 16 and electronic supplementary material, table S7. While we visualize the full model, we only discuss the main parameters of interest (please see the highlighted panels (*a*–*c*) in figure 16). In terms of our main hypotheses, the posterior distribution for the three-way interaction effects of session, group and ANT (executive, orienting, alerting) overlaps with zero, which suggests mostly an invariance to art versus non-art training in terms of executive, orienting, alerting measures. This suggests similar sensitivity of attentional functioning to art versus non-art training group and across pre- versus post-sessions.

We also performed a model comparison analysis, with results detailed in electronic supplementary material, figure S26.

### 4.4. Discussion

Experiment 3 showed that the impact of the training session (pre- versus post-training) on measures of executive functions (executive, orienting, alerting) did not vary by group type (art versus non-art). These findings do not provide support for our hypothesis regarding the interaction between the executive functions of attention and post-art training effects. Therefore, we cannot conclude that art training makes a distinct contribution to improvements in executive functions of attention. Instead, we can conclude that art and non-art knowledge development have an equivalent impact on executive functions.

At this point, we want to highlight and discuss the most obvious limitation of Experiment 3. In hindsight, it was foolhardy and possibly naïve to expect that a relatively short intervention of any kind might impact core dimensions of executive functions. This may be because executive functions are fairly stable over time and hard to shape by brief interventions [128]. At the same time, we are empirical scientists, and we are aware of many other brief interventions that have been aimed at executive functions, such as mindfulness training [53,54]. As such, although we may have been expecting too much from a brief intervention, we still feel that the clear and transparent reporting of this work can help in the longer term in the development of knowledge by guiding the design of new experiments via the use of our data and materials, as well as in meta-analyses and data syntheses. Furthermore, given the problems with the widespread under-reporting of null results, which leads to publication bias [129] and the file-drawer problem [130], we feel it is far better for scientific progress and future understanding to make null results available, especially when the data collection plan and analyses have been pre-registered in advance [65].

## 5. General discussion

Across three pre-registered experiments, we examined the ways in which art knowledge training impacts subsequent aesthetic judgements of artworks and executive functions of attention. We showed that art knowledge training led to greater ratings of understanding, artistic skill, aesthetic preference and affective judgements for trained rather than untrained artworks (Experiment 1 and Experiment 2), and as a function of larger art training doses than smaller doses (Experiment 2). We also found that the aesthetic judgements generalized to unseen artworks as a function of how similar the unseen artwork was to the training materials. Specifically, the effects generalized to unseen artworks produced by the same artist (Experiment 1 and Experiment 2) and by a different artist as long as both artists shared a similar artistic style (Experiment 2). Furthermore, we found an invariance to the type of content being trained (art versus non-art) in terms of reaction times measures of alerting, orienting, executive functions of attention (Experiment 3). Taken together, these findings suggest that (i) art knowledge training shapes subsequent aesthetic judgements; (ii) acquiring art knowledge through training encourages generalizability effects to new contexts, which are largely similar to the training material; and (iii) improvements in executive functions of attention are equally explained by art knowledge training and non-art knowledge training.

### 5.1. Implications for expertise development and generalizability effects

The current findings provide support for the hypothesis that aesthetic judgements can be shaped through learning and accumulation of knowledge. Given that the art knowledge training enhanced the ratings for understanding the meaning and artistic skill of artworks, this suggests that understanding the meaning and artistic qualities of artworks contributes to a perceiver’s aesthetic experience. Along with previous research on contextual art knowledge, such as artworks’ titles, these findings demonstrate that art knowledge aids meaningful interpretation of artworks and enhances aesthetic appraisal [57–63,131,132].

Moreover, we found that the impact of training on evaluative judgements generalizes to unseen, but similar artwork used in training, suggesting that art knowledge generalizability effects depend on the artistic style similarity between the trained and novel artwork. Previous research on skill learning described the generalization effects of training in terms of near transfer (transfer to similar settings) and far transfer (transfer to different settings) [35,36]. Our findings can be interpreted as evidence for near transfer rather than far transfer. That is, the art training improvements occurred near to (rather than far beyond) the content of the acquired knowledge. In the current case, learning about Sorolla’s Impressionist art enhanced subsequent aesthetic judgements about novel, unseen artworks produced by the same Impressionist artist (Sorolla) and by a different Impressionist artist (Merritt-Chase), but not about Gauguin’s Post-Impressionist art. This suggests that similarity-based transfer for art knowledge is not linked to the same artist, but extends to different artists if they share similar artistic style features, such as line, shape, colour, etc. Taken together, these ideas highlight the relevance of the theoretical frameworks according to which transfer depends on the similarity between knowledge learned initially and its later applications [133,134].

### 5.2. Implications for art training optimal dosage

The current findings further emphasize that art knowledge dose is important for shaping aesthetic judgements of artworks and for the generalization of acquired knowledge to novel contexts. Specifically, approximately 16 minutes and approximately 22 minutes of art knowledge training increased not just art understanding and art appreciation for trained materials, but also fostered generalization effects of training to similar and different artists if they shared comparable artistic style features. These findings have implications for art galleries and museum curators worldwide. Since art museums aim to educate and communicate art knowledge to diverse audiences [80,135], knowing the required art knowledge dose to facilitate aesthetic judgements about the featured artworks and generalization of art knowledge to similar artistic styles offers valuable evidence-based information for individuals and organizations engaged in arts education.

### 5.3. Implications for executive functions supporting art knowledge development

Our results do not provide evidence to suggest that art knowledge training represents a unique contributor to improvements in attentional functioning. Given that our art knowledge training was created to guide participants’ viewing through different elements of the pictorial scene, from global visual scanning to detail-focused, one future direction could be examining the impact of art knowledge training on visuospatial skills measured by visual search tasks. In this sense, Wiesmann & Ishai [136] have shown that art training can improve object recognition and the visual search strategies to recognize objects. Moreover, medical researchers have found that after the art training medical students had better scores on visual observational skills, and analytical thinking skills [52,137,138], suggesting that art knowledge training can impact the domain-general cognitive abilities.

As both art and non-art training included comprehensive narratives and rich visual images and descriptions, it is plausible that they might have equally contributed to some improvements in executive functions. For example, both training types contained in-depth explanations of visual scenes and meaningful interpretations of the content which might have contributed to attentional functioning. This possibility is supported by recent work using eye-tracking, which demonstrates that overt attention in visual scenes is primarily and involuntary guided by rich semantic content of the scene [139,140]. Together this suggests that meaningful scenes are salient and may capture and guide visual attention.

At the same time, the current results may reflect a combination of practice effects with rich visual and semantic content saliency. As the current design did not use a passive control group, it is impossible to rule out possible practice effects. In this respect, previous research has conceptualized practice effects in cognitive tasks, as improved participants’ performance strategies to carry out a task [141]. Evidence examining the robustness, stability and reliability of attentional network test (ANT) has shown small practice effects over 10 testing subsequent sessions [142], suggesting that ANT performance may be affected by repeated evaluation with the same test.

Executive functions of attention are a part of what we call general human intelligence, alongside long-term and working memory, processing speed, reasoning and solving problems [143]. Given that the art training was created considering rich declarative knowledge and concepts, the possibility of improvements is not limited to just attentional functioning; they potentially include visuospatial working memory or divergent thinking abilities. In that regard, previous studies have demonstrated that artists compared with non-artists show an advantage to non-verbal IQ tests, mental rotation tasks, visual memory, analytical abilities measured by embedded figures tests [20,44,47,144,145]. Therefore, it could be that a combination of perception-to-action interventions (e.g. drawing, painting) with art knowledge training might be suited to measuring improvements on different measures of cognitive functioning.

In addition, in our final experiment, we chose not to include a no-training control group, as such a control group would not have helped us to address our primary research question. Instead, we compared art-based training with a non-art-based type of training (rather than a passive control condition). The benefit of our approach is that one can assess whether any training effects are art-specific rather than a result of more general experience. Based on our research design, however, we cannot comment on whether our findings would be similar or different when compared with a passive control condition and it remains for future research to clarify this matter.

### 5.4. Applied implications

The current work has implications for designing future art educational programmes in schools and art museums, as it informs the extent to which learning about art modulates subsequent aesthetic judgements and the degree to which knowledge transfer occurs to new contexts. This study demonstrates that art training interventions are successful in different laboratory settings using online individual participation and in-person classroom participation. This provides empirical support for future art interventions that different experimental settings can facilitate learning and generalization of knowledge to new contexts with relatively short, pre-recorded intervention materials.

At the same time, those designing interventions should be cautious about the possible reach that an art intervention may have, in terms of generalizability. That is not to say that a number of health and social benefits might emerge from engaging in an art intervention [26,146–148], but we did not focus on these measures here. However, what we do show is that transfer effects are limited to the same artist or similar artistic styles, as well as the larger training doses that we studied (i.e. > 16 minutes). Since art knowledge expertise development is underrepresented in psychological research and art educational programmes are affected by frequent economic cuts [149–152], it would be wise to set expectations accordingly when planning interventions.

### 5.5. Limitations and constraints on generality

As indicated by Simons *et al*. [153], it is important to highlight constraints on generality of our findings. We show similarity-based generalization effects of training to both unseen artworks produced by the same Impressionist artist (Sorolla) and a different Impressionist artist (Merritt-Chase), but not a different Post-Impressionist artist (Gauguin). Our results can be interpreted in the context of representational art; future studies might extend the investigation to include abstract art and more cultural variation. While our results reflect an art knowledge training that was visual and auditory in nature, we cannot exclude the possibility that different generalization effects might be observed when using a single sensory modality training, such as visual modality only. Future studies might help to clarify that aspect. Furthermore, given that our art and non-art knowledge training reflects a semantic form of expertise, our findings should be interpreted specifically within that context and with caution when considering the visual-motor expertise acquired by studio artists. While both forms of expertise share some features, they also involve distinct elements. Consequently, further research is necessary to explore these different types of expertise in greater depth. In addition, our research design cannot disentangle whether different components of our art knowledge training (e.g., historical, visual characteristics) had distinct impacts on judgements of artworks. Future investigations would be required to assess this possibility.

Next, we turn to consider an alternative explanation for the results in Experiments 1 and 2 that relates to a ‘mere exposure’ effect [154]. A mere exposure argument would suggest that simply seeing repeated stimuli, rather than the training intervention that we used, could account for our results by making familiar stimuli more likeable. Since our experimental design did not have a condition that only showed repeated stimuli instead of the intervention material that we used, our design cannot rule out this possibility. With this said, we find it rather unlikely that passive viewing of artworks would lead to the exact same effects as an approximately 20-minutes documentary that walks people through a crafted narrative that outlines the rich history, context and style of Sorolla’s artworks. Nonetheless, an interesting avenue for future research would be to directly compare a narrative intervention like the one we used with a mere exposure condition.

Our findings from Experiments 1 and 2 should be viewed considering the longstanding tradition in empirical aesthetics research of using self-reported aesthetic judgements. However, a promising direction for future research could involve using memory-based paradigms or objective-performance based measures instead of self-reported responses. In addition, given the prior work on executive function and cultural influences in aesthetic appreciation [155,156], future research might investigate how art knowledge training and culture impact executive function.

In addition, our study is firmly grounded in broader theoretical frameworks, such as learning, expertise development, generalization effects and dose–response relationships. Nevertheless, future research may benefit from using theoretical models specifically focused on aesthetics to motivate their predictions. Finally, given our sample sizes, it is important to acknowledge that there may be smaller effects that we were unable to detect with a reasonable degree of confidence. Future research should, therefore, consider conducting follow-up studies specifically designed to detect smaller effect sizes.

## Data Availability

All the data, stimuli and analysis scripts for each experiment are available online via the Open Science Framework [157].
